# Comparative effectiveness of different dual task mode interventions on cognitive function in older adults with mild cognitive impairment or dementia: a systematic review and network meta-analysis

**DOI:** 10.1007/s40520-025-03016-5

**Published:** 2025-04-30

**Authors:** Yuqing Hao, Yajie Zhao, Huanhuan Luo, Lanying Xie, Huixiu Hu, Chao Sun

**Affiliations:** 1 Department of Nursing, Beijing Hospital, National Center of Gerontology, Institute of Geriatric Medicine, Chinese Academy of Medical Science, No. 1 Da Hua Road, DongDan, Beijing, 100730 People’s Republic of China; 2 Beijing Hospital, National Center of Gerontology, Institute of Geriatric Medicine, Chinese Academy of Medical Sciences and Peking Union Medical College, Beijing, People’s Republic of China; 3 Department of Cardiology, Beijing Hospital, National Center of Gerontology, Institute of Geriatric Medicine, Chinese Academy of Medical Science, Beijing, People’s Republic of China; 4 School of Nursing, Beijing University of Traditional Chinese Medicine, Beijing, People’s Republic of China

**Keywords:** Cognitive function, Combined interventions, Dementia, Dual task, Mild cognitive impairment, Network meta-analysis

## Abstract

**Objectives:**

To evaluate and compare the effects of different dual task interventions on cognitive function in older adults with cognitive impairment or dementia.

**Methods:**

We searched eight databases, including PubMed, Cochrane Library, and EMBASE, to obtain studies exclusively comprising randomized controlled trials on dual task interventions in individuals aged 60 and older with mild cognitive impairment or dementia, up to July 28, 2024. Study quality was evaluated using the Cochrane Risk of Bias Tool. Analyses included pairwise meta-analyses via Review Manager 5.4 and network meta-analyses via Stata 14.0.

**Results:**

A total of 32 RCTs involving 2370 participants were included. Dual cognitive task training had the most significant impact on global cognition (SUCRA = 79.2%, mean rank = 1.6) and motor-cognitive dual task training was the only dual task intervention with a notable improvement in executive function (SMD = 1.53, 95% CI 0.06–3.01). For physical function, dual motor task training was most effective, improving gait performance (SMD = 0.34), muscle strength (SMD = 0.28), and balance (SMD = 0.90). Motor-cognitive dual task training demonstrated the greatest effectiveness in enhancing activities of daily living (SMD = 1.50) and quality of life (SMD = 1.20), while reducing depressive symptoms (SMD = − 0.96).

**Conclusions:**

Dual cognitive task training is the most effective dual task intervention for enhancing global cognition. Motor-cognitive dual task training is the only dual task mode that significantly improves executive cognition.

**Supplementary Information:**

The online version contains supplementary material available at 10.1007/s40520-025-03016-5.

## Introduction

As global life expectancy rises and the aging population expands, the incidence of age-related cognitive impairment among older adults is escalating. It is currently estimated that over 55 million people worldwide suffer from dementia, and over 10 million new cases are identified each year [1]. According to the World Health Organization (WHO), dementia significantly impairs cognitive function in older adults, reducing their ability to perform activities of daily living (ADL), and is a major contributor to disability and reliance among this population globally [2]. Progressive cognitive decline contributes to diminished physical abilities, such as slower walking speed and reduced lower-limb muscle strength, which in turn results in gait disturbances and decreased mobility in patients with dementia [3]. Therefore, cognitive decline often coexists with physical frailty. Frailty is characterized by the cumulative decline of multiple physiological systems, manifested by unintentional weight loss, self-reported exhaustion, weakness, slow walking speed, and low physical activity [4]. This combination of cognitive decline and physical frailty is referred to as cognitive frailty [5]. Moreover, cognitive deterioration frequently coincides with impairments in ADL, adversely affecting the quality of life (QOL) for both individuals with dementia and their caregivers, while also placing significant economic and caregiving burdens on families and society [6, 7]. Mild cognitive impairment (MCI) constitutes a transitional phase between normal age-related cognitive decline and dementia, marked by significant cognitive deficits that have not yet met the diagnostic threshold for dementia [8]. During this stage, individuals display noticeable cognitive impairments in complex functional tasks, yet they retain the ability to function independently in daily activities [9]. This period is crucial for implementing preventive interventions to halt the progression to dementia.

Older adults with MCI and dementia face a broad array of challenges, including cognitive deficits, physical functional impairments, emotional disturbances, etc. Despite this, current pharmacological treatments for dementia, such as acetylcholinesterase inhibitors like donepezil and memantine, offer only modest symptomatic relief and do not halt disease progression, with safety concerns and limited efficacy remaining significant challenges [10]. Given the limited high-quality evidence supporting pharmacological treatments for dementia, exploring effective non-drug interventions to slow cognitive deterioration is essential. Evidence suggests that cognitive and motor activity interventions represent effective non-pharmacological approaches to attenuating cognitive decline in individuals with MCI [11]. The study of Küster et al. [12] elucidate that they may impact cognitive functions through distinct biological mechanisms: physical activities elevate brain-derived neurotrophic factor (BDNF) levels which are essential for neuroplasticity, whereas cognitive training is linked to reductions in the neurotoxic metabolite 3-HK, potentially alleviating neuronal stress.

Dual tasking consists of combining two different single tasks, involves performing two tasks, either sequentially or concurrently, leveraging concepts of motor skill acquisition or task relevance to efficiently handle multiple tasks. According to Campos-Magdaleno et al. [13], this study considers three distinct dual task pairings: motor-cognitive, dual motor, and dual cognitive. To distinguish ‘dual task’ from the execution of a complex single task, it is imperative that the dual task meets two primary criteria: (1) each component task can be independently performed and quantitatively assessed; and (2) each task possesses distinct objectives [14].

Systematic reviews indicate that dual task training offers greater benefits compared to single cognitive or physical task training in reducing age-related cognitive decline [15]. Given the variety of dual task modalities available, it is crucial to discern which specific mode is most effective for improving cognitive and functional aspects in older adults with cognitive impairments. Moreover, cognitive-motor dual task interventions, as a novel approach, raise the question of whether their effects differ from those of traditional interventions that combine two cognitive or physical tasks under the same conditions. Consequently, we conducted this network meta-analysis to synthesize existing evidence and determine the most effective dual task interventions for enhancing cognitive function in older adults with MCI or dementia, thereby supporting evidence-based clinical decision-making.

## Methods

This systematic review and network meta-analysis followed the PRISMA Extension for Network Meta-Analyses (PRISMA-NMA) guidelines to ensure a thorough and transparent presentation of the methodology and findings [16]. The study protocol was proactively registered at PROSPERO, registration number CRD42024550565, before the initiation of data extraction and analysis. As this study was a meta-analysis, ethical approval and consent to participate were not applicable.

### Data sources and search strategy

Searches were conducted in both English and Chinese databases, including PubMed, Cochrane Library, EMBASE, CINAHL, China National Knowledge Infrastructure, Wanfang Data, Chinese Biomedical Literature Database and VIP, from their inception to July 28, 2024. Our search strategy incorporated both subject words and free words. For instance, in searching for “Dementia”, we used the subject word “dementia” along with free words like “Cognitive Decline” and “Cognitive Impairment”. To ensure the inclusion of all pertinent studies, we manually searched the reference lists of systematic reviews related to the topic published in the last five years. The detailed search strategy is presented in Supplementary Appendix 1.

### Selection criteria

Initially, duplicate records were removed using EndNote X9 software (Clarivate Analytics, Philadelphia, PA, USA). Following this, two researchers (YH and YZ) independently screened the titles and abstracts. Studies that met the inclusion criteria were then subjected to a detailed assessment by both reviewers. In cases of disagreement or discrepancies, a third author was consulted to achieve consensus. The specific inclusion criteria were as follows: (1) Participants: Older adults aged 60 and older who have been clinically diagnosed with MCI or dementia. (2) Interventions: Each intervention program must employ one of the following dual task modes: dual cognitive task training (involving two cognitive task interventions), dual motor task training (involving two motor task interventions), or motor-cognitive dual task training (involving one motor and one cognitive task intervention), whether these tasks are performed simultaneously or sequentially. In order to include as many trials as possible, this study did not set limits on the type, dose, duration, intensity or frequency of the intervention. (3) Comparison: Control interventions may include single-task physical or cognitive activity, usual care, waiting lists, health education, placebo training or dual task modes similar to interventions. (4) Outcomes: The primary outcome focused on global cognition, secondary outcomes included executive function, memory function, depressive symptoms, ADL, gait performance, muscle strength, QOL, and balance. Studies were included if they reported either the primary outcome (global cognition) or at least one of the secondary outcomes. (5) Study design: only randomized controlled trials were included. Articles meeting the following criteria will be excluded: (1) Studies published in languages other than English or Chinese. (2) Studies involving additional interventions with potential cognitive therapeutic effects, such as cognitive-behavioral therapy or reminiscence therapy. (3) Conference abstracts, reviews, study protocols, and letters to the editor. (4) Studies were excluded if they had incomplete data that precluded comprehensive analysis or assessment of outcomes. (5) When multiple articles reported results from the same study, only the most recently published article was included.

### Data extraction

Data were independently extracted by two reviewers using a predefined standardized form, with discrepancies resolved by consensus. Extracted information included: (1) Country and year of publication; (2) Sample characteristics, including type of disease, sample size, mean age, gender distribution, baseline cognitive function of participants. (3) Intervention details, including components of physical or cognitive interventions, combination forms, duration, frequency, and total intervention length. (4) The control measures used for comparison. (5) Information on outcomes, including global cognition, executive function, memory function, depressive symptoms, activities of daily living, gait performance, muscle strength, QOL and balance performance. We assessed baseline comparability across included studies and excluded any studies that did not clearly report baseline characteristics. If data were not reported in the articles, efforts were made to contact the original authors to obtain the missing information. When standard deviations (SDs) were not reported, they were derived from available standard errors (SEs), confidence intervals (CIs), t or p values. We also reached out to the authors via email to obtain any missing data. Additionally, when data were presented only graphically but not reported numerically, we utilized GetData Graph Digitizer version 2.20 to extract the necessary information from the graphs. In instances where both unadjusted and adjusted data were provided, the analysis incorporated the adjusted data to ensure the accuracy and relevance of our findings.

### Risk of bias

The risk of bias in the included studies was evaluated by two independent reviewers using the Cochrane Collaboration’s risk of bias tool. Given that it was not possible to blind participants during the implementation of the motor or cognitive interventions, we assessed only the other 6 categories of risk bias included in the study, which included random sequence generation, allocation concealment, blinding of the outcome assessor, incomplete data, selective reporting, and other sources of bias (such as baseline imbalance and funding bias) [17].

### Data analysis

Pairwise meta-analyses were conducted using Review Manager 5.4 provided by the Cochrane Collaboration. Network meta-analyses were carried out using the Network package in STATA (Version 14.0; StataCorp, College Station, TX, USA), employing a random-effects model based on a frequency-domain framework [18]. The mean difference and standard deviation of the required data were extracted or calculated from the included studies, and the standardized mean difference (SMD) along with the 95% confidence interval (CI) were calculated to summarize the effect size [19]. For studies using multiple neuropsychological tests to assess the same outcomes, we selected the most commonly utilized test as the representative measure to ensure consistency and comparability across studies. Heterogeneity was assessed using the Q test and I^2^, with a Q test of p < 0.1 or I^2^ ≥ 50%. In such cases, a random-effects model was employed; otherwise, a fixed-effects model was used.

Network diagrams were employed to assess the structure of the intervention network and potential biases. In these diagrams, each node represents an intervention, with the node size reflecting the sample size. Lines connecting the nodes, with thickness representing the number of studies, illustrate direct comparisons between interventions [20]. Inconsistency within the network was assessed using a global inconsistency test. A nonsignificant result (p > 0.05) indicated that the direct and indirect evidence were coherent, thereby justifying the use of a consistency model for further analysis [21]. Local inconsistency was further evaluated using the node-splitting method, which compares direct and indirect estimates for specific comparisons within the network [22]. The network geometry is depicted in a network graph, and the relative effect estimates of pairwise comparisons are summarized in a league table. Loop inconsistency tests were performed to verify the consistency of direct and indirect evidence across various loops [23]. Using the Surface Under the Cumulative Ranking Curve (SUCRA), we quantified the impact of various interventions on cognitive function in elderly patients with mild cognitive impairment or dementia. Values on the SUCRA scale range from 0 to 100%, with higher percentages indicating a higher probability that an intervention is the most effective. Funnel plots were employed to assess publication bias, visualizing the symmetry of effect sizes relative to their standard errors across studies [22].

## Results

### Study selection

**Fig. 1:**
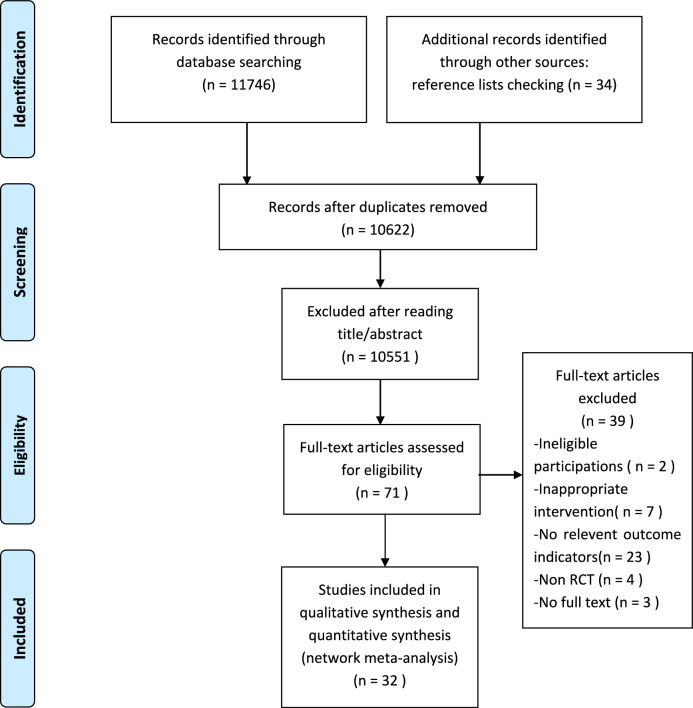
The specific PRISMA-NMA flow diagram

Database searches initially identified 11,746 records, with an additional 34 articles obtained from the reference lists of systematic reviews related to the topic published in the last five years. After deduplication, 10,622 records remained. Subsequent screening of titles and abstracts excluded 10,551 records, leaving 71 articles for full-text review. Of these, 39 records were further excluded due to various reasons: ineligible participants (n = 2), inappropriate interventions (n = 7), lack of relevant outcome indicators (n = 23), non-RCTs (n = 4), and absence of full text (n = 3). Ultimately, 32 RCTs were included in the study. The specific PRISMA-NMA flow diagram is shown in Fig. 1.

### The overview of all included studies

Table 1 presents the characteristics of the included studies. All dual task intervention combinations included in the study included a cognitive-motor dual task intervention (n = 16, primarily a combination of aerobic and memory tasks), a dual motor task intervention (n = 11, primarily a combination of aerobic and strength training), and a dual cognitive task intervention (n = 5, primarily a combination of memory training and executive function training). Intervention durations varied from 4 to 26 weeks; most studies (n = 20, 62.5%) conducted interventions that lasted no less than 12 months, with the most common duration being 12 weeks (n = 9, 28.13%). Session lengths ranged from 10 to 100 min, with 30 min sessions being the most frequent (n = 15, 46.9%). The interventions were conducted between once and seven times per week, with biweekly sessions being the most common frequency (n = 12, 37.5%). Included studies were listed in Supplementary Appendix 2.

**Table 1:** The characteristics of the included studies

The main characteristics of included studies											
References	Country	Type of disease	Dual task interventions								Comparison measures
			Sample size (Gender distribution, Male/Female)	Age (Mean ± SD)	Dual task mode	Intervention design	Duration (min/session)	Frequency (times/week)	Outcome measures	Global Cognition (Baseline vs Post-intervention, Mean ± SD)	
Tappen et al. [44]	USA	Dementia	21 (NR)	84.3 ± 7.53	Motor-cognitive dual task training	Self-paced assisted walking combined with conversation	30	3	GP	NR	AC: Self-paced assisted walking
Yoon et al. [27]	South Korea	Dementia	15 (NR)	77.9 ± 7.5	Motor-cognitive dual task training	Cycling exercise and sequential memory recall tasks	30	3	GC, balance, depression, memory	50.6 ± 34.7 vs 39.8 ± 33.0 (7 Minute Screening)	AC: Cognitive training
Fiatarone Singh et al. [48]	Australia	MCI	27 (NR)	70.1 ± 6.7	Motor-cognitive dual task training	Progressive resistance training and cognitive training	100	2	GC, EC, memory	8.02 ± 1.15 vs 6.26 ± 1.15 (ADAS-Cog)	PC: Sham cognitive and exercise interventions
Chu et al. [30]	China	Dementia	52 (26/26)	82 ± 6.08	Motor-cognitive dual task training	Listening to popular music and performing motor movements to the music	30	2	GC, depression,	12.80 ± 6.15 vs 15.72 ± 6.53 (MMSE)	PC: Usual care
Law et al. [36]	Australia	MCI	43 (16/27)	73.68 ± 6.8	Motor-cognitive dual task training	Performing simulated functional tasks following specific patterns of a movement sequence	30	1	GC, EC, memory, ADL	50.65 ± 11.77 vs 62.23 ± 13.72 (NCSE)	AC: Cognitive training
Hughes et al. [32]	USA	MCI	10 (2/8)	78.5 ± 7.1	Motor-cognitive dual task training	Interactive video gaming	90	1	GC, GP	25.55 ± 6.24 vs 29.41 ± 5.48 (CAMCI)	AC: Health education
Hagovská et al. [50]	Slovak	MCI	40 (22/18)	68 ± 4.4	Motor-cognitive dual task training	Cognitive training with CogniPlus and balance training	30	7	GC, balance, QOL	25.97 ± 2.57 vs 26.97 ± 2.21 (MMSE)	AC: Balance training
Shimizu et al. [25]	Japan	MCI	35 (6/29)	74.90 ± 4.29	Motor-cognitive dual task training	Repetitive rhythmic movements with a percussion instrument (Naruko clappers) to music	50	1	EC, balance, GP, MS	NR	AC: Single-task training
Zheng and Chen [46]	China	Dementia	18 (3/15)	81.74 ± 5.79	Motor-cognitive dual task training	Kinect-based somatosensory game	10	5	GC, depression	14.06 ± 6.66 vs 16.78 ± 7.18 (MMSE)	PC: Usual care
Juniarti et al. [33]	Indonesia	Dementia	45 (10/35)	62.4 ± 3.5	Motor-cognitive dual task training	Reading aloud activity and low-impact aerobic exercise	60	3	GC	22.49 ± 2.75 vs 24.96 ± 3.31 (MMSE)	PC: Waiting-list
Liu et al. [38]	China	MCI	16 (4/12)	74.6 ± 6.1	Motor-cognitive dual task training	Exergaming-Based Tai Chi	50	3	GC, EC, GP	22.6 ± 2.5 vs 24.5 ± 0.38 (MoCA)	PC: Usual care
Kuo et al. [51]	China	MCI	11 (1/10)	78 ± 8.15	Motor-cognitive dual task training	Walking while performing a motor task	45	3	EC, memory, GP	NR	AC: Single walking
			9 (1/8)	80 ± 7.04	Motor-cognitive dual task training	Walking while performing a cognitive task	45	3	EC, memory, GP	NR	
Embon-Magal et al. [31]	Israel	MCI	28 (11/17)	81.16 ± 8.23	Motor-cognitive dual task training	Thinking in motion (TIM) intervention: a combined motor-cognitive co-dependent intervention	40	3	GC, GP	3.96 ± 1.20 vs 3.22 ± 1.35 (CogniFit)	AC: Computerized cognitive training
Menengiç et al. [40]	Turkey	Dementia	10 (3/7)	77.7 ± 5.29	Motor-cognitive dual task training	Motor-cognitive dual task training via telerehabilitation	40	4	GC, balance, ADL, Depression	18.2 ± 3.55 vs 21.8 ± 2.85 (MMSE)	PC: Usual care
Baldimtsi et al. [29]	Greece	MCI	28 (7/21)	66.07 ± 10.04	Motor-cognitive dual task training	Complete 20 simple numerical calculations (single-digit additions and subtractions) while cycling	30	2 or 3	GC, memory, EC, ADL	28.26 ± 1.48 vs 28.17 ± 2.01 (MMSE)	PC: Usual care
Ayed et al. [28]	Tunisia	MCI	15 (5/10)	67.13 ± 3.04	Motor-cognitive dual task training	Combined aerobic and cognitive games training	30	2	GC, EC, memory, depression, QOL	26.13 ± 0.35 vs 27.13 ± 0.83 (MMSE)	AC: Reading
Vreugdenhil et al. [26]	Australia	Dementia	20 (11/9)	73.5	Dual motor task training	Daily home-based exercises plus walking	30	7	GC, balance, MS, ADL, depression	22.9 ± 5.0 vs 23.9 ± 5.0 (MMSE)	PC: Usual care
Telenius et al. [54]	Norway	Dementia	87 (24/63)	87.3 ± 7.0	Dual motor task training	Lower limb strength plus balance exercises	50	2	GC, ADL, GP, balance, MS, depression, QOL	15.5 ± 0.6 vs 14.4 ± 0.6 (MMSE)	AC: Light physical activity
Bossers et al. [47]	Netherlands	Dementia	37 (8/29)	85.7 ± 5.1	Dual motor task training	Aerobic plus strength training	30	4	GC, memory, balance, MS	15.81 ± 4.30 vs 17.16 ± 4.33 (MMSE)	AC: Social visit
Toots et al. [55]	Sweden	Dementia	93 (23/70)	85.1 ± 7.1	Dual motor task training	Lower limb strength plus balance exercises	45	2	ADL, balance	NR	AC: Seated control activities
Lamb et al. [52]	UK	Dementia	329 (195/134)	76.9 ± 7.9	Dual motor task training	Aerobic plus strength training	60	2	GC, QOL, ADL	21.4 ± 9.6 vs 25.2 ± 12.3 (ADAS-cog)	PC: Usual care
Langoni et al. [35]	Brazil	MCI	26 (6/20)	72.6 ± 7.8	Dual motor task training	Aerobic plus strength training	60	2	GC, MS, balance, GP	21.9 ± 4.8 vs 25.0 ± 4.7 (MMSE)	PC: Usual care
Sanders et al. [53]	Netherlands	Dementia	46 (25/21)	72.6 ± 7.8	Dual motor task training	Outdoor walking plus lower limb strength exercises	30	3	GC, EC, memory, GP, MS, balance	21.37 ± 3.94 vs 20.43 ± 4.78 (MMSE)	NR
			45 (23/22)	71.9 ± 7.9	Dual motor task training	Flexibility exercise and board games	30	3		19.54 ± 4.77 vs 18.74 ± 5.87 (MMSE)	
Mak et al. [39]	Australia	MCI/Dementia	113 (66/47)	86.0	Dual motor task training	Pneumatic resistance training (using HUR Health and Fitness Equipment) plus balance exercise	30	2	GC, MS, balance, GP	23.5 ± 4.1 vs 24.1 ± 4.1 (MMSE)	PC: Usual care
Uysal et al. [45]	Turkey	MCI	12 (10/2)	73.5 ± 3.21	Dual motor task training	Aerobic exercise training combined with lower limb strengthening	30	3	GC, balance, MS, depression, QOL	20.92 ± 0.9 vs 22.75 ± 1.06 (MMSE)	AC: Lower limb strengthening
Papatsimpas et al. [42]	Greece	Dementia	57 (21/36)	76.82 ± 5.73	Dual motor task training	Combined aerobic plus resistance exercise	30	5	GC, EC, memory, ADL	74.04 ± 7.42 vs 79.25 ± 6.46 (ACE-R)	PC: Usual care
Guzel and Can [49]	Turkey	Dementia	11 (1/10)	82.3 ± 6.7	Dual motor task training	Combined aerobic and balance exercise	40	2	GC, memory, MS	14.63 ± 3.10 vs 17.00 ± 3.43 (MMSE)	AC: Aerobic exercise
Kawashima et al. [34]	Japan	Dementia	16 (NR)	85.1 ± 5.4	Dual cognitive task training	Reading aloud plus arithmetic calculation	20	6	EC	NR	PC: Usual care
Herrera et al. [24]	France	MCI	11 (6/5)	75.09 ± 1.97	Dual cognitive task training	Memory training and attention training	60	2	Memory	NR	AC: Cognitive stimulation
Lee et al. [37]	China	Dementia	7 (1/6)	77.7 ± 6.07	Dual cognitive task training	Memory enhancement training plus executive function training	30	2	GC, depression, ADL, memory	17.00 ± 3.58 vs 19.67 ± 5.20 (MMSE)	PC: Waiting-list
Shyu et al. [43]	China	Dementia	15 (10/5)	82 ± 5	Dual cognitive task training	Memory training plus central executive system training	30	1	GC	22 ± 3 vs 24 ± 4 (MMSE)	AC: Health education
Nousia et al. [41]	Greece	MCI	15 (8/7)	75.73 ± 4.48	Dual cognitive task training	Cognitive training using the RehaCom Cognitive Therapy Software plus language exercises with paper and pencil	60	2	GC, memory	22.73 ± 1.44 vs 23.67 ± 1.59 (MoCA)	PC: Usual care

*NR* Not reported, *GP* Gait performance, *AC* Active control, *GC* Global cognition, *EC* Executive cognition, *ADAS-Cog* Alzheimer’s Disease Assessment Scale-Cognitive Subscale, *PC* Passive control, *ADL* Activities of daily living, *NCSE* Neurobehavioral Cognitive Status Examination, *GP* Gait performance, *CAMCI* Computer Assessment of Mild Cognitive Impairment, *QOL* Quality of life, *MS* Muscle strength, *MMSE* Mini-Mental State Examination, *MoCA* The Montreal Cognitive Assessment

### Risk of bias

In the reviewed studies, 4 studies [24–27] were identified as having high concerns due to incomplete data, potentially impacting their outcomes. 19 studies [28–46] demonstrated certain concerns resulting from unclear reporting of one or more pieces of information. The remaining 9 studies [47–55] were evaluated and classified as low concern. The overall risk of bias assessment across all included studies is summarized in Supplementary Appendix 3, and the detailed risk of bias assessments for each individual study are provided in Supplementary Appendix 4.

### Traditional pairwise meta-analysis

In traditional pairwise meta-analyses, motor-cognitive dual task training, dual motor task training, and dual cognitive task training all showed significant effects in global cognition and memory function in older adults with MCI or dementia compared with control interventions. Dual cognitive task training emerged as the most potent, enhancing global cognition significantly (n = 3, sample sizes 37 vs 36, SMD = 1.15, 95% CI 0.65 to 1.66). Motor-cognitive dual task training was also significant in improving executive function (SMD = 1.40, 95% CI 0.44 to 2.36) and depressive symptoms (SMD = − 0.95, 95% CI − 1.44 to − 0.46), whereas there were no significant differences from controls in ADL (SMD = 1.47, 95% CI − 0.05 to 2.99), gait performance (SMD = − 0.15, 95% CI − 0.43 to 0.14) and balance (SMD = 0.23, 95% CI − 0.08 to 0.54). Dual motor task training had significantly more benefits in improving physical function, with significant effects in improving ADL (SMD = 0.42, 95% CI 0.29 to 0.55), gait performance (SMD = 0.35, 95% CI 0.02 to 0.67), muscle strength (SMD = 0.72, 95% CI 0.37 to 1.07) and balance (SMD = 0.47, 95% CI 0.31 to 0.62) in older adults with cognitive impairments. More details are shown in Supplementary Appendix 5.

### Network meta-analysis

**Fig. 2:**
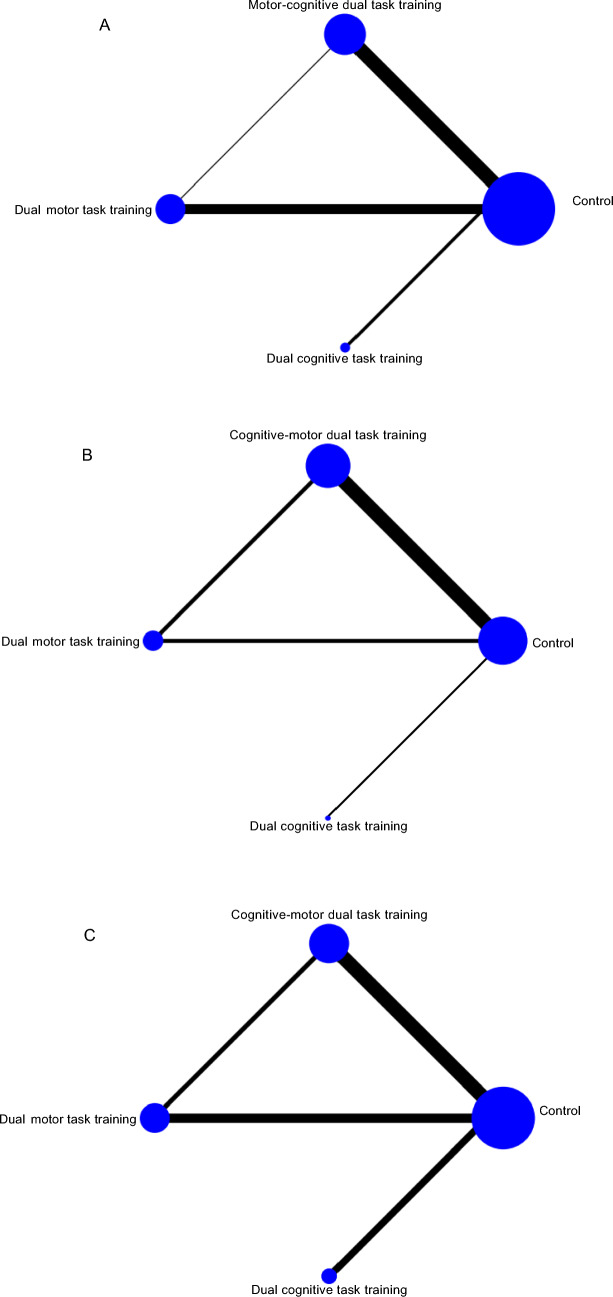
The network diagram for cognitive function: **A** global cognition, **B** executive function, and **C** memory function. The node size correlates with the number of participants randomly assigned to each intervention, and the line thickness between two nodes reflects the quantity of RCTs comparing those interventions

We conducted a network meta-analysis to evaluate the effects of various dual task intervention methods on eight outcome measures. These measures span 4 domains: cognitive domains (including global cognition, executive function, and memory function), physical function (encompassing ADL, gait performance, muscle strength, and balance), depressive symptoms and QOL. The network diagram for cognitive domains is presented in Fig. 2, while the diagrams for the remaining eight outcomes are in Supplementary Appendix 6. Of all the studies, the control group had the largest sample size (890 participants), followed by dual motor task training (738 participants), motor-cognitive dual task training (392 participants), and dual cognitive task training (37 participants). Loop inconsistency tests supported the overall consistency of the network, confirming coherent results across key cognitive outcomes such as global cognition, memory function, and executive function.

#### Cognitive domains

**Fig. 3:**
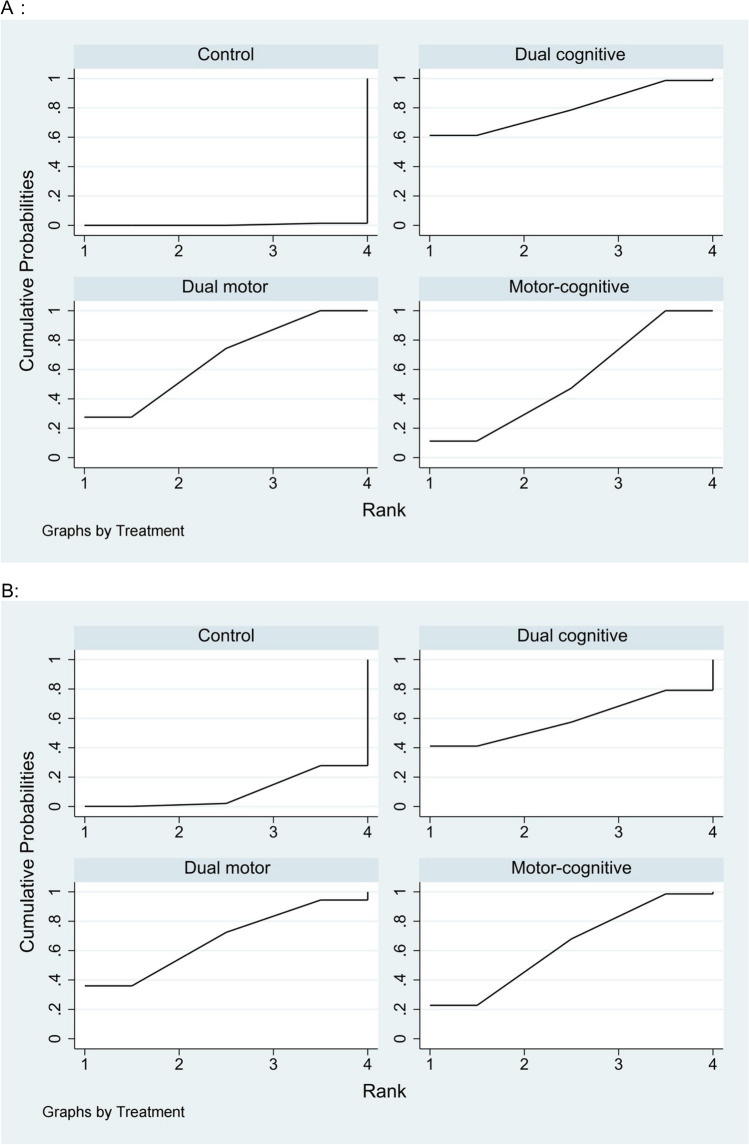

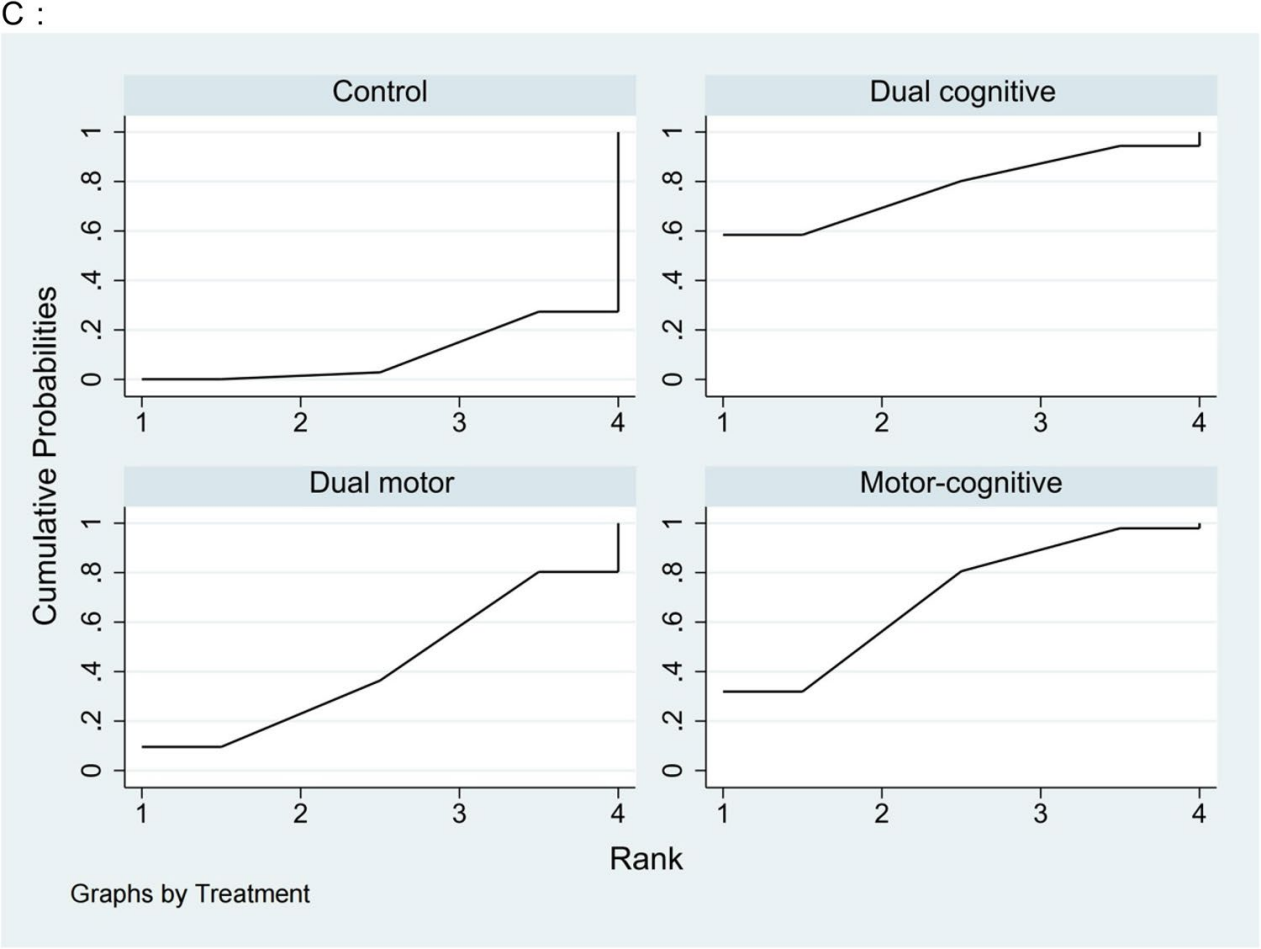
The probability ranking for all interventions: **A** global cognition, **B** executive function, and **C** memory function. Abbreviations: Dual cognitive, dual cognitive task training; Dual motor, dual motor task training; Motor-cognitive, motor-cognitive dual task training

Figure 2 displays the network diagram for cognitive domains, including outcomes for global cognition, executive function, and memory function. The instruments used for measuring global cognition included the Mini-Mental State Examination (MMSE), Montreal Cognitive Assessment (MoCA), Alzheimer’s Disease Assessment Scale-Cognitive subscale (ADAS-Cog), Computerized Assessment of Mild Cognitive Impairment (CAMCI), 7 Minute Screening, Neurobehavioral Cognitive Status Examination (NCSE) and Addenbrooke’s Cognitive Examination-Revised (ACE-R). Table 2 presents the estimated effects of each intervention on cognitive function as derived from the network meta-analysis. The network meta-analysis revealed that motor-cognitive dual task training (SMD = 0.73, 95% CI 0.31 to 1.16), dual motor task training (SMD = 0.86, 95% CI 0.38 to 1.35), and dual cognitive task training (SMD = 1.09, 95% CI 0.12 to 2.06) all significantly improve global cognition compared to the control group. Figure 3 illustrates the SUCRA for each intervention type in enhancing cognitive function. Results highlight that dual cognitive task training is the most effective intervention for improving global cognition in elderly patients with MCI and dementia (SUCRA = 79.2%, mean rank = 1.6), followed by dual motor task training (SUCRA = 67.4%, mean rank = 2.0), motor-cognitive dual task training (SUCRA = 52.8%, mean rank = 2.4), and the control group (SUCRA = 0.6%, mean rank = 4). Inconsistency within the network meta-analysis was evaluated using loop-specific tests, revealing minimal inconsistency in the assessment of global cognition with an inconsistency factor of 0.21 (95% CI 0.00 to 2.03) and heterogeneity (τ^2^) of 0.376, suggesting coherent direct and indirect comparisons. Funnel plot analysis for publication bias showed no significant asymmetry, indicating the findings are robust and free from publication bias. Further details on these analyses are provided in Supplementary Appendices 9 and 10.

**Table 2:** Results of the comparative effectiveness for (A) global cognition, (B) executive function, and (C) memory function are presented

A			
**Dual cognitive task training**			
0.23 (− 0.86, 1.32)	**Dual motor task training**		
0.36 (− 0.70, 1.42)	0.13 (− 0.48, 0.74)	**Motor-cognitive dual task training**	
**1.09 (0.12, 2.06)**	**0.86 (0.38, 1.35)**	**0.73 (0.31, 1.16)**	**Control**

B			
**Dual motor task training**			
0.19 (− 2.11, 2.49)	**Motor-cognitive dual task training**		
0.05 (− 4.57, 4.67)	− 0.14 (− 4.41, 4.13)	**Dual cognitive task training**	
1.72 (− 0.57, 4.02)	**1.53 (0.06, 3.01)**	1.67 (− 2.33, 5.68)	**Control**

C			
**Dual cognitive task training**			
0.35 (− 1.78, 2.49)	**Motor-cognitive dual task training**		
0.88 (− 1.36, 3.12)	0.53 (− 0.98, 2.03)	**Dual motor task training**	
1.44 (− 0.36, 3.24)	1.08 (− 0.07, 2.23)	0.56 (−0.78, 1.89)	**Control**

League table showing the relative effect estimates of different dual task interventions on A: global cognition; B: executive cognition; C: memory function. Positive value favors the column; all statistically significant effects are shown in bold

Each measurement is presented as a SMD with a 95% CI. For each measurement, a negative SMD indicates a preference for the lower-right intervention; a positive SMD favors the upper-left intervention. Results that are statistically significant are highlighted in bold

Regarding executive function, motor-cognitive task training significantly outperformed the control group (SMD = 1.53, 95% CI 0.06 to 3.01), while dual motor task training (SMD = 1.72, 95% CI − 0.57 to 4.02) and dual cognitive task training (SMD = 1.67, 95% CI − 2.33 to 5.68) did not show significant differences compared to the control group. Moreover, the result showed that, no significant differences were observed in any pairwise comparisons for memory cognition.

#### Physical function

Dual motor task training demonstrates considerable efficacy within the sphere of physical function. Dual motor task training demonstrated superior improvements in gait performance compared to both the control group (SMD = 0.34, 95% CI 0.11 to 0.56) and motor-cognitive dual task training (SMD = 0.48, 95% CI 0.18 to 0.78), and achieved the highest probability of ranking best (SUCRA = 63.8%, mean rank = 1.7). Dual motor task training also emerged as the only intervention to manifest a significant improvement in muscle strength over the control group (SMD = 0.28, 95% CI 0.38 to 1.45). Furthermore, it was the only intervention that exhibited an improvement in balance compared to the control group (SMD = 0.90, 95% CI 0.08 to 1.71). Notably, studies on dual cognitive task training were excluded from the analysis above as they did not report on these specific physical function outcomes. For ADL, motor-cognitive dual task training was the only intervention that demonstrated a significant improvement compared to the control group, with a substantial effect size (SMD = 1.50, 95% CI 0.50 to 2.50).

#### Depressive symptoms and quality of life

The network for depressive symptoms consisted of nine studies comparing 4 treatments. In addressing depressive symptoms, motor-cognitive dual task training significantly surpassed the control group, achieving a notable reduction in symptom severity (SMD = − 0.96, 95% CI − 1.51 to − 0.41), which suggests its potential utility in managing depressive conditions among the elderly. Regarding QOL, motor-cognitive dual task training also demonstrated more pronounced improvements compared to both the control and dual motor task training (SMD = 1.20 and 1.31, respectively). Forest plots of the pairwise network meta-analysis results for all outcome measures are detailed in Supplementary Appendix 7, with the SUCRAs and the league tables presented in Supplementary Appendix 8.

## Discussion

### Main findings and interpretation

Our findings reveal that all evaluated dual task interventions are promising for improving global cognition relative to the control group. Consistent with previous research, dual task training has been demonstrated to confer benefits on cognitive functions in populations with dementia or MCI, particularly in attention [56]. The results suggest that dual cognitive task training is potentially the most effective for improving global cognition, followed by dual motor task training and motor-cognitive dual task training. Dual cognitive task training typically involves memory training combined with other training such as arithmetic calculation, attention, executive function, and language exercises, using both digital tools like RehaCom Cognitive Therapy Software and traditional methods like paper and pencil exercises. It is hypothesized that such training fosters cognitive and brain reserves, which confer increased resilience against neuropathological impacts [57]. Dual cognitive task training, characterized by repetitive and targeted cognitive exercises, is likely to induce more profound neuroplastic changes compared to dual motor tasks and motor-cognitive dual tasks. These neuroplastic changes enhance neural efficiency, thereby more directly influencing global cognition [41]. Given these findings, healthcare providers can implement dual cognitive task interventions for older adults with MCI or dementia to improve their global cognition. In community settings, healthcare providers can offer flexible and accessible interventions by integrating dual cognitive training into daily activities. For example, community centers could facilitate activities that combine memory exercises with group storytelling or collaborative puzzles, which can be easily implemented by community health nurses or caregivers. These activities not only enhance cognitive function but also promote social interaction and mental well-being among older adults. On the other hand, in long-term care settings, where residents often have more severe cognitive impairments and limited mobility, structured and guided interventions may be more appropriate. Digital dual cognitive training programs can be introduced to guide patients through structured exercises, providing a standardized and scalable approach to dual cognitive task interventions.

Furthermore, only motor-cognitive dual task training showed significant improvements in executive functions, as verified by previous systematic review [15]. This effect can be attributed to the fact that motor-cognitive dual task training necessitates the simultaneous execution of cognitive tasks and physical activities. This dual engagement likely activates both motor and cognitive control networks within the brain, stimulating regions involved in task coordination and problem-solving, including the prefrontal cortex, which is pivotal for executive functions. In terms of enhancing memory function, our analysis did not detect significant differences between any dual task intervention and the control group. However, a systematic review has pointed out that computerized cognitive training (CCT) can improve specific memory domains in MCI participants, such as verbal, visual, and working memory [58]. This study employed a variety of cognitive tasks targeting multiple cognitive domains, which did not meet our inclusion criteria since we only accepted studies explicitly involving two cognitive tasks and excluded those with three or more. This highlights that interventions incorporating a broader range of cognitive tasks may be more beneficial for managing and improving memory functions. Future research should explore the effectiveness of multifaceted cognitive task interventions in enhancing memory performance.

Psychological symptoms and functional status are also recognized as critical goals in dementia care [59]. Consequently, it is also essential to examine the effects of dual task interventions on these important non-cognitive outcomes. Our study demonstrates that dual motor task training significantly improves physical function in older adults with cognitive impairments, evidenced by marked improvements in gait performance, muscle strength, and balance compared to control conditions. Dual motor tasks may be particularly effective in improving physical function due to their emphasis on repetitive functional movements [60]. Such movements directly target specific motor patterns, reinforcing neuromuscular coordination and enhancing muscle memory. By repeatedly practicing functional tasks, such as walking or balancing exercises, dual motor tasks strengthen the neural pathways associated with these activities, leading to more efficient motor control and better physical performance. Furthermore, consistent with recent research [61], our findings did not observe significant improvements in physical function such as gait performance and balance from motor-cognitive dual task training. This may be due to the fact that improvements in balance primarily depend on specific motor exercises designed to enhance sensorimotor integration and neuromuscular coordination. However, another systematic review [62] reported that motor-cognitive dual task training led to improvements in gait performance among older adults with cognitive impairment. This could be attributed to their exclusive focus on simultaneous tasks, in contrast to our study which included both simultaneous and sequential tasks. Furthermore, our results suggest that motor-cognitive dual task training may be particularly effective in improving ADL and quality of life. This could be attributed to the inherent requirements of ADLs, which typically necessitate the simultaneous execution of multiple tasks demanding both high motor and cognitive resources, and motor-cognitive dual task training could enhance the neural and motor coordination needed for these multitasking demands [63].

While dual motor task training or motor-cognitive dual task training may not match the efficacy of dual cognitive task training in improving global cognition, they significantly enhance overall functional abilities, especially in terms of non-cognitive outcomes. Integrating physical exercises into dual task training regimes not only supports cognitive rehabilitation but also substantially increases functional capacity through the enhancement of strength and balance. These benefits are critical in mitigating the risk of falls and combating other age-related deteriorations in functional abilities, thereby extending considerable health advantages to the elderly population.

### Strengths and limitations

This study is the first network meta-analysis to assess the efficacy of various dual task mode interventions on cognitive functions in older adults with mild cognitive impairment or dementia, employing a rigorous and comprehensive search strategy limited to randomized controlled trials for the highest level of evidence. Compared to previous systematic reviews on the effectiveness of dual task training for cognitively impaired older adults, our study further categorized dual task types, applied more stringent inclusion and exclusion criteria, and analyzed the comparative effects between different dual task combined interventions. However, some limitations should be noted. Firstly, the study’s scope was restricted to articles published in English and Chinese, potentially introducing selection bias due to the exclusion of studies in other languages, additional sources, and grey literature. Secondly, variations in intervention intensity, duration, and frequency across included studies may have introduced heterogeneity and potential confounding, limiting the ability to precisely compare effectiveness across intervention types. Additionally, for certain outcome categories (global cognition, QOL, and ADLs), the sample size in one study [52] may have dominated the network meta-analysis, necessitating a cautious interpretation of these results. Finally, the limited sample sizes in original studies on dual cognitive task training precluded further subgroup analysis to identify optimal intervention frequencies and durations.

This study is the first network meta-analysis to assess the efficacy of various dual task mode interventions on cognitive functions in older adults with mild cognitive impairment or dementia, employing a rigorous and comprehensive search strategy limited to randomized controlled trials for the highest level of evidence. Compared to previous systematic reviews on the effectiveness of dual task training for cognitively impaired older adults, our study further categorized dual task types, applied more stringent inclusion and exclusion criteria, and analyzed the comparative effects between different dual task combined interventions. However, some limitations should be noted. The study’s scope was restricted to articles published in English and Chinese, potentially introducing selection bias due to the exclusion of studies in other languages, additional sources, and grey literature. For certain outcome categories (global cognition, QOL, and ADLs), the sample size in one study [52] may have dominated the network meta-analysis, necessitating a cautious interpretation of these results. Additionally, the limited sample sizes in original studies on dual cognitive task training precluded further subgroup analysis to identify optimal intervention frequencies and durations.

## Conclusion

Our findings underscore the critical role of dual task interventions, particularly dual cognitive tasks, in the strategic management of MCI or dementia within community settings. Dual motor tasks are particularly effective in improving physical function, whereas motor-cognitive dual tasks are most beneficial for enhancing quality of life, ADL, as well as alleviating depression symptoms. Healthcare providers should, therefore, tailor these approaches based on specific patient needs to promote overall health in individuals at preclinical and clinical stages of dementia. To support the effective implementation and dissemination of the relevant interventions, the next step could be to consider the establishment of multidisciplinary teams comprising, for example, neurologists, physiotherapists and geriatric specialist nurses. Pilot projects funded by health policymakers could evaluate the feasibility of these interventions across different community settings, thereby providing crucial data to refine and optimize care programs. In addition, health authorities could consider incorporating dual task training into health guidelines for dementia care to ensure that these beneficial interventions receive the attention and funding needed for widespread adoption. Such strategic integration is expected to greatly enhance the management of dementia and potentially aid its development in community settings.

## Supplementary Information

Below is the link to the electronic supplementary material.

Supplementary file1 (DOCX 2498 KB)

## Data Availability

No datasets were generated or analysed during the current study.
