# Supplementary material for: Comparative effectiveness of different dual task mode interventions on cognitive function in older adults with mild cognitive impairment or dementia: a systematic review and network meta-analysis
Source: Aging Clin Exp Res. 2025 Apr 30;37(1):139. doi: 10.1007/s40520-025-03016-5 (PMC12043736; doi:10.1007/s40520-025-03016-5)
Supplement: Supplementary file 1 — Supplementary file1 (DOCX 2498 KB) [file 40520_2025_3016_MOESM1_ESM.docx]

**Content**

**Supplementary Appendix 1 -- Search Strategy**

**Supplementary Appendix 2 -- The list for the included studies**

**Supplementary Appendix 3 -- The overall risk of bias for all included studies**

**Supplementary Appendix 4 -- The risk of bias for each study**

**Supplementary Appendix 5 -- Results of the traditional pairwise meta-analysis**

**Supplementary Appendix 6 -- The network meta-analysis diagrams for the remaining outcomes**

**Supplementary Appendix 7 -- Results of the loop-specific inconsistency tests**

**Supplementary Appendix 8 -- Funnel plot for all outcomes**

**Supplementary Appendix 9 -- Pairwise network meta-analysis results**

**Supplementary Appendix 10 -- The ranking of effects for the remaining outcomes：the SUCRAs and the League tables**

**Supplementary Appendix 1 -- Search Strategy: e.g., PubMed.**

| Database | Search Strategy |
| --- | --- |
| PubMed | Search: (((((("Cognitive Dysfunction"[Mesh]) OR "Dementia"[Mesh]) OR (((((((((((((((((((((((((((((((((((((((((Cognitive Dysfunctions[Title/Abstract]) OR (Dysfunction, Cognitive[Title/Abstract])) OR (Dysfunctions, Cognitive[Title/Abstract])) OR (Cognitive Impairments[Title/Abstract])) OR (Cognitive Impairment[Title/Abstract])) OR (Impairment, Cognitive[Title/Abstract])) OR (Impairments, Cognitive[Title/Abstract])) OR (Cognitive Disorder[Title/Abstract])) OR (Cognitive Disorders[Title/Abstract])) OR (Disorder, Cognitive[Title/Abstract])) OR (Disorders, Cognitive[Title/Abstract])) OR (Mild Cognitive Impairment[Title/Abstract])) OR (Cognitive Impairment, Mild[Title/Abstract])) OR (Cognitive Impairments, Mild[Title/Abstract])) OR (Impairment, Mild Cognitive[Title/Abstract])) OR (Impairments, Mild Cognitive[Title/Abstract])) OR (Mild Cognitive Impairments[Title/Abstract])) OR (Cognitive Decline[Title/Abstract])) OR (Cognitive Declines[Title/Abstract])) OR (Decline, Cognitive[Title/Abstract])) OR (Declines, Cognitive[Title/Abstract])) OR (Mental Deterioration[Title/Abstract])) OR (Deterioration, Mental[Title/Abstract])) OR (Deteriorations, Mental[Title/Abstract])) OR (Mental Deteriorations[Title/Abstract])) OR (Cogniti* Complain*[Title/Abstract])) OR (Cogniti* Problem[Title/Abstract])) OR (Cogniti* issue[Title/Abstract])) OR (Alzheimer*[Title/Abstract])) OR (Dement*[Title/Abstract])) OR (Aphasia, Primary Progressive[Title/Abstract])) OR (Primary Progressive Nonfluent Aphasia[Title/Abstract])) OR (Creutzfeldt-Jakob Syndrome[Title/Abstract])) OR (Dementia, Vascular[Title/Abstract])) OR (CADASIL[Title/Abstract])) OR (Diffuse Neurofibrillary Tangles with Calcification[Title/Abstract])) OR (Frontotemporal Lobar Degeneration[Title/Abstract])) OR (Primary Progressive Nonfluent Aphasia[Title/Abstract])) OR (Huntington Disease[Title/Abstract])) OR (Kluver-Bucy Syndrome[Title/Abstract])) OR (Lewy Body Disease[Title/Abstract]))) AND (("Aged"[Mesh]) OR ((((Old[Title/Abstract]) OR (Elder*[Title/Abstract])) OR (Aging[Title/Abstract])) OR (Aged[Title/Abstract])))) AND ((((("Cognitive Training"[Mesh]) OR "Exercise"[Mesh]) OR "Exergaming"[Mesh]) OR "Resistance Training"[Mesh]) OR ((((((((((((((((((((((((((((((((((((((((((((Exercis*[Title/Abstract]) OR (physical*[Title/Abstract])) OR (train*[Title/Abstract])) OR (motor Activit*[Title/Abstract])) OR (Motor Training[Title/Abstract])) OR (Motor Exercise*[Title/Abstract])) OR (Motor Stimulation[Title/Abstract])) OR (Resistance Exercise*[Title/Abstract])) OR (Strength Training[Title/Abstract])) OR (Strength Exercise*[Title/Abstract])) OR (Balanc*[Title/Abstract])) OR (Brain training[Title/Abstract])) OR (Computer* training[Title/Abstract])) OR (Memory training[Title/Abstract])) OR (Attention training[Title/Abstract])) OR (Cognitive task[Title/Abstract])) OR (Cognitive-task[Title/Abstract])) OR (Cognitive Exercise*[Title/Abstract])) OR (Cognitive Rehabilitation[Title/Abstract])) OR (Cognitive stimulation[Title/Abstract])) OR (Cognitive Method[Title/Abstract])) OR (Cognitive Program*[Title/Abstract])) OR (Cognitive Treatment[Title/Abstract])) OR (Memory Intervention[Title/Abstract])) OR (Motor-cognitive[Title/Abstract])) OR (Multi-task[Title/Abstract])) OR (Multitask*[Title/Abstract])) OR (Divided attention[Title/Abstract])) OR (cognitive-motor[Title/Abstract])) OR (Cognitive motor[Title/Abstract])) OR (motor cognitive[Title/Abstract])) OR (Multi-component[Title/Abstract])) OR (Multicomponent[Title/Abstract])) OR (Multidomain[Title/Abstract])) OR (Multi-domain[Title/Abstract])) OR (Multimodal[Title/Abstract])) OR (Multi-modal[Title/Abstract])) OR (Combin*[Title/Abstract])) OR (Dualtask[Title/Abstract])) OR (Dual task[Title/Abstract])) OR (Dual-task[Title/Abstract])) OR (Combined Modality Therapy[Title/Abstract])) OR (Simultaneous[Title/Abstract])) OR (Synergistic[Title/Abstract])))) AND (((((Cognitive function[Title/Abstract]) OR (Cognitive ability[Title/Abstract])) OR (Cognitive performance[Title/Abstract])) OR (Neurocognitive function[Title/Abstract])) OR (Mini-mental state examination[Title/Abstract]))) AND ((((Placebo[Title/Abstract]) OR (randomized[Title/Abstract])) OR (randomly[Title/Abstract])) OR (randomized controlled trial[Publication Type])) Sort by: Most Recent |

**Supplementary Appendix 2 -- The list for the included studies**

1. Ayed, I.B., Aouichaoui, C., Ammar, A., Naija, S., Tabka, O., Jahrami, H., Trabelsi, K., Trabelsi, Y., El Massioui, N., El Massioui, F., 2024. Mid-term and long-lasting psycho-cognitive benefits of bidomain training intervention in elderly individuals with mild cognitive impairment. Eur. J. Investig. Health Psychol. Educ. 14, 284–298.

2. Baldimtsi, E., Mouzakidis, C., Karathanasi, E.M., Verykouki, E., Hassandra, M., Galanis, E., Hatzigeorgiadis, A., Goudas, M., Zikas, P., Evangelou, G., Papagiannakis, G., Bellis, G., Kokkotis, C., Tsatalas, T., Giakas, G., Theodorakis, Y., Tsolaki, M., 2023. Effects of virtual reality physical and cognitive training intervention on cognitive abilities of elders with mild cognitive impairment. J. Alzheimers Dis. Rep. 7, 1475–1490.

3. Bossers, W.J.R., van der Woude, L.H.V., Boersma, F., Hortobágyi, T., Scherder, E.J.A., van Heuvelen, M.J.G., 2015. A 9-Week Aerobic and Strength Training Program Improves Cognitive and Motor Function in Patients with Dementia: A Randomized, Controlled Trial. Am. J. Geriatr. Psychiatry Off. J. Am. Assoc. Geriatr. Psychiatry 23, 1106–1116.

4. Chu, H., Yang, C.Y., Lin, Y., Ou, K.L., Lee, T.Y., O’Brien, A.P., Chou, K.R., 2014. The impact of group music therapy on depression and cognition in elderly persons with dementia: a randomized controlled study. Biol. Res. Nurs. 16, 209–217.

5. Embon-Magal, S., Krasovsky, T., Doron, I., Asraf, K., Haimov, I., Gil, E., Agmon, M., 2022. The effect of co-dependent (thinking in motion [TIM]) versus single-modality (CogniFit) interventions on cognition and gait among community-dwelling older adults with cognitive impairment: A randomized controlled study. BMC Geriatr. 22, 720.

6. Fiatarone Singh, M.A., Gates, N., Saigal, N., Wilson, G.C., Meiklejohn, J., Brodaty, H., Wen, W., Singh, N., Baune, B.T., Suo, C., Baker, M.K., Foroughi, N., Wang, Y., Sachdev, P.S., Valenzuela, M., 2014. The study of mental and resistance training (SMART) study—resistance training and/or cognitive training in mild cognitive impairment: A randomized, double-blind, double-sham controlled trial. J. Am. Med. Dir. Assoc. 15, 873–880.

7. Guzel, I., Can, F., 2024. The effects of different exercise types on cognitive and physical functions in dementia patients: A randomized comparative study. Arch. Gerontol. Geriatr. 119, 105321.

8. Hagovská, M., Olekszyová, Z., 2016. Impact of the combination of cognitive and balance training on gait, fear and risk of falling and quality of life in seniors with mild cognitive impairment. Geriatr. Gerontol. Int. 16, 1043–1050.

9. Herrera, C., Chambon, C., Michel, B.F., Paban, V., Alescio-Lautier, B., 2012. Positive effects of computer-based cognitive training in adults with mild cognitive impairment. Neuropsychologia 50, 1871–1881.

10. Hughes, T., Flatt, J., Fu, B., Butters, M., Cc, C., M, G., 2014. Interactive video gaming compared with health education in older adults with mild cognitive impairment: A feasibility study. Int. J. Geriatr. Psychiatry 29.

11. Juniarti, N., Al’Adawiyah Mz, I., Sari, C.W.M., Haroen, H., 2021. The Effect of Exercise and Learning Therapy on Cognitive Functions and Physical Activity of Older People with Dementia in Indonesia. J. Aging Res. 2021, 6647029.

12. Kawashima, R., Okita, K., Yamazaki, R., Tajima, N., Yoshida, H., Taira, M., Iwata, K., Sasaki, T., Maeyama, K., Usui, N., Sugimoto, K., 2005. Reading aloud and arithmetic calculation improve frontal function of people with dementia. J. Gerontol. A. Biol. Sci. Med. Sci. 60, 380–384.

13. Kuo, H.T., Yeh, N.C., Yang, Y.R., Hsu, W.C., Liao, Y.Y., Wang, R.Y., 2022. Effects of different dual task training on dual task walking and responding brain activation in older adults with mild cognitive impairment. Sci. Rep. 12, 8490.

14. Lamb, S.E., Sheehan, B., Atherton, N., Nichols, V., Collins, H., Mistry, D., Dosanjh, S., Slowther, A.M., Khan, I., Petrou, S., Lall, R., DAPA Trial Investigators, 2018. Dementia And Physical Activity (DAPA) trial of moderate to high intensity exercise training for people with dementia: randomised controlled trial. BMJ 361, k1675.

15. Langoni, C., Resende, T., Barcellos, A., Cecchele, B., Ms, K., Tdn, S., Jn, da R., Ts, D., Igds, F., Cha, S., 2019. Effect of exercise on cognition, conditioning, muscle endurance, and balance in older adults with mild cognitive impairment: A randomized controlled trial. J. Geriatr. Phys.

16. Law, L., Barnett, F., Yau, M., Ma, G., 2014. Effects of functional tasks exercise on older adults with cognitive impairment at risk of alzheimer’s disease: A randomised controlled trial. Age Ageing 43.

17. Lee, G.Y., Yip, C.C.K., Yu, E.C.S., Man, D.W.K., 2013. Evaluation of a computer-assisted errorless learning-based memory training program for patients with early Alzheimer’s disease in Hong Kong: a pilot study. Clin. Interv. Aging 8, 623–633.

18. Liu, C.L., Cheng, F.Y., Wei, M.J., Liao, Y.Y., 2022. Effects of Exergaming-Based Tai Chi on Cognitive Function and Dual-Task Gait Performance in Older Adults With Mild Cognitive Impairment: A Randomized Control Trial. Front. Aging Neurosci. 14, 761053.

19. Mak, A., Delbaere, K., Refshauge, K., Henwood, T., Goodall, S., Clemson, L., Hewitt, J., Taylor, M.E., 2022. Sunbeam Program Reduces Rate of Falls in Long-Term Care Residents With Mild to Moderate Cognitive Impairment or Dementia: Subgroup Analysis of a Cluster Randomized Controlled Trial. J. Am. Med. Dir. Assoc. 23, 743-749.e1.

20. Menengi Ç, K.N., Yeldan, İ., Çınar, N., Şahiner, T., 2022. Effectiveness of motor-cognitive dual-task exercise via telerehabilitation in alzheimer’s disease: An online pilot randomized controlled study. Clin. Neurol. Neurosurg. 223, 107501.

21. Nousia, A., Pappa, E., Siokas, V., Liampas, I., Tsouris, Z., Messinis, L., Patrikelis, P., Manouilidou, C., Dardiotis, E., Nasios, G., 2023. Evaluation of the efficacy and feasibility of a telerehabilitation program using language and cognitive exercises in multi-domain amnestic mild cognitive impairment. Arch. Clin. Neuropsychol. Off. J. Natl. Acad. Neuropsychol. 38, 224–235.

22. Papatsimpas, V., Vrouva, S., Papathanasiou, G., Papadopoulou, M., Bouzineki, C., Kanellopoulou, S., Moutafi, D., Bakalidou, D., 2023. Does therapeutic exercise support improvement in cognitive function and instrumental activities of daily living in patients with mild alzheimer’s disease? A randomized controlled trial. Brain Sci. 13, 1112.

23. Sanders, L.M.J., Hortobágyi, T., Karssemeijer, E.G.A., Van der Zee, E.A., Scherder, E.J.A., van Heuvelen, M.J.G., 2020. Effects of low- and high-intensity physical exercise on physical and cognitive function in older persons with dementia: a randomized controlled trial. Alzheimers Res. Ther. 12, 28.

24. Shimizu, N., Umemura, T., Matsunaga, M., Hirai, T., 2018. Effects of movement music therapy with a percussion instrument on physical and frontal lobe function in older adults with mild cognitive impairment: A randomized controlled trial. Aging Ment. Health 22.

25. Shyu, Y.I.L., Lin, C.C., Kwok, Y.T., Shyu, H.Y., Kuo, L.M., 2022. A community-based computerised cognitive training program for older persons with mild dementia: A pilot study. Australas. J. Ageing 41, e82–e93.

26. Tappen, R.M., Roach, K.E., Applegate, E.B., Stowell, P., 2000. Effect of a combined walking and conversation intervention on functional mobility of nursing home residents with alzheimer disease. Alzheimer Dis. Assoc. Disord. 14, 196–201.

27. Telenius, E., Engedal, K., Bergland, A., 2015. Long-term effects of a 12 weeks high-intensity functional exercise program on physical function and mental health in nursing home residents with dementia: A single blinded randomized controlled trial. BMC Geriatr. 15.

28. Toots, A., Littbrand, H., Lindelöf, N., Wiklund, R., Holmberg, H., Nordström, P., Lundin-Olsson, L., Gustafson, Y., Rosendahl, E., 2016. Effects of a High-Intensity Functional Exercise Program on Dependence in Activities of Daily Living and Balance in Older Adults with Dementia. J. Am. Geriatr. Soc. 64, 55–64.

29. Uysal, İ., Başar, S., Aysel, S., Kalafat, D., Büyüksünnetçi, A.Ö., 2023. Aerobic exercise and dual-task training combination is the best combination for improving cognitive status, mobility and physical performance in older adults with mild cognitive impairment. Aging Clin. Exp. Res. 35, 271–281.

30. Vreugdenhil, A., Cannell, J., Davies, A., Razay, G., 2012. A community-based exercise programme to improve functional ability in people with alzheimer’s disease: A randomized controlled trial. Scand. J. Caring Sci. 26, 12–19.

31. Yoon, J.E., Lee, S.M., Lim, H.S., Kim, T.H., Jeon, J.K., Mun, M.H., 2013. The effects of cognitive activity combined with active extremity exercise on balance, walking activity, memory level and quality of life of an older adult sample with dementia. J. Phys. Ther. Sci. 25, 1601–1604.

32. Zheng J., Chen X., 2018. The effect of Kinect-based somatosensory game on patients with dementia. J. Nurs. Sci. 33, 5–9.

**Supplementary Appendix 3 -- The overall risk of bias for all included studies**

**Supplementary Appendix 4 -- The risk of bias for each study**

**Supplementary Table 5 -- Results of the traditional pairwise meta-analysis**

| Comparison | Outcomes | No. of studies | SMD | 95% CI | P | heterogeneity test | | |
| --- | --- | --- | --- | --- | --- | --- | --- | --- |
|  |  |  |  |  |  | I^2^ | X^2^ | p |
| Motor-cognitive dual-task training versus Control | Global cognition | 13 | 0.72 | 0.35, 1.08 | 0.001 | 80% | 59.24 | ＜0.001 |
|  | Memory function | 6 | 1.05 | 0.28, 1.81 | 0.007 | 87% | 38.61 | ＜0.001 |
|  | Executive cognition | 7 | 1.40 | 0.44, 2.36 | 0.004 | 92% | 75.23 | ＜0.001 |
|  | Depressive symptoms | 5 | -0.95 | -1.44, -0.46 | 0.001 | 61% | 10.13 | 0.04 |
|  | ADL | 3 | 1.47 | -0.05, 2.99 | 0.06 | 93% | 29.27 | ＜0.001 |
|  | Gait performance | 6 | -0.15 | -0.43, 0.14 | 0.31 | 0 | 2.33 | 0.80 |
|  | Balance | 4 | 0.23 | -0.08, 0.54 | 0.15 | 36% | 4.69 | 0.20 |
| Dual motor task training versus Control | Global cognition | 9 | 0.41 | 0.29, 0.53 | 0.001 | 91% | 90.02 | ＜0.001 |
|  | Memory function | 4 | 1.00 | 0.70, 1,29 | 0.001 | 95% | 56.38 | ＜0.001 |
|  | Depressive symptoms | 3 | -0.19 | -0.44, 0.07 | 0.16 | 74% | 7.55 | 0.02 |
|  | ADL | 5 | 0.42 | 0.29, 0.55 | 0.001 | 89% | 35.46 | 0.001 |
|  | Gait performance | 4 | 0.35 | 0.02, 0.67 | 0.04 | 57% | 7.03 | 0.07 |
|  | Muscle strength | 6 | 0.72 | 0.37, 1.07 | 0.001 | 71% | 17.34 | 0.004 |
|  | Quality of life | 3 | -0.11 | -0.26, 0.05 | 0.18 | 49% | 3.90 | 0.14 |
|  | Balance | 7 | 0.47 | 0.31, 0.62 | 0.001 | 98% | 243.72 | ＜0.001 |
| Dual cognitive task training versus Control | Global cognition | 3 | 1.15 | 0.65, 1.66 | 0.001 | 12% | 2.28 | 0.32 |
|  | Memory function | 3 | 0.79 | 0.23, 1.36 | 0.006 | 90% | 19.40 | ＜0.001 |

**Supplementary Appendix 6 -- The network meta-analysis diagrams for the remaining outcomes**

1. Depressive symptoms
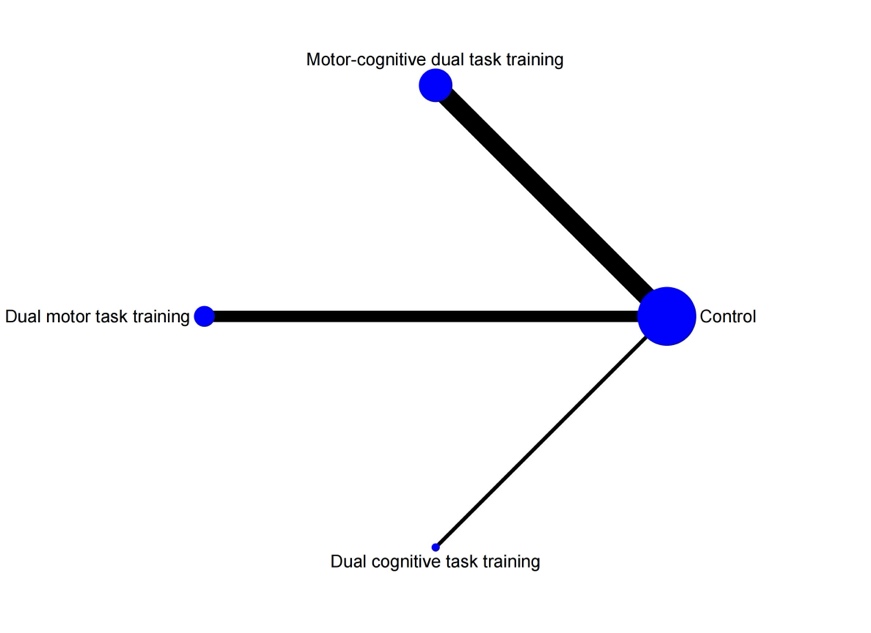

2. ADL


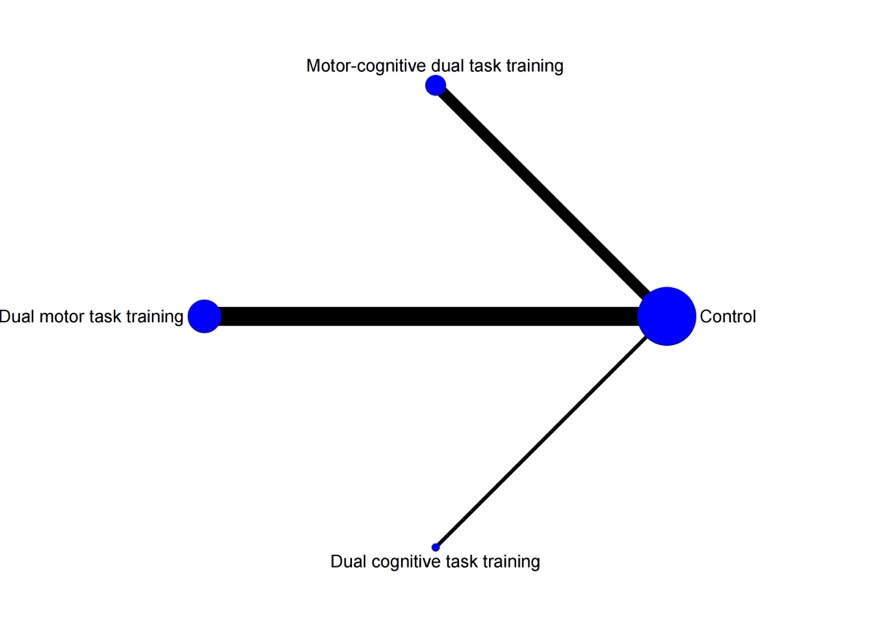


1. Gait performance


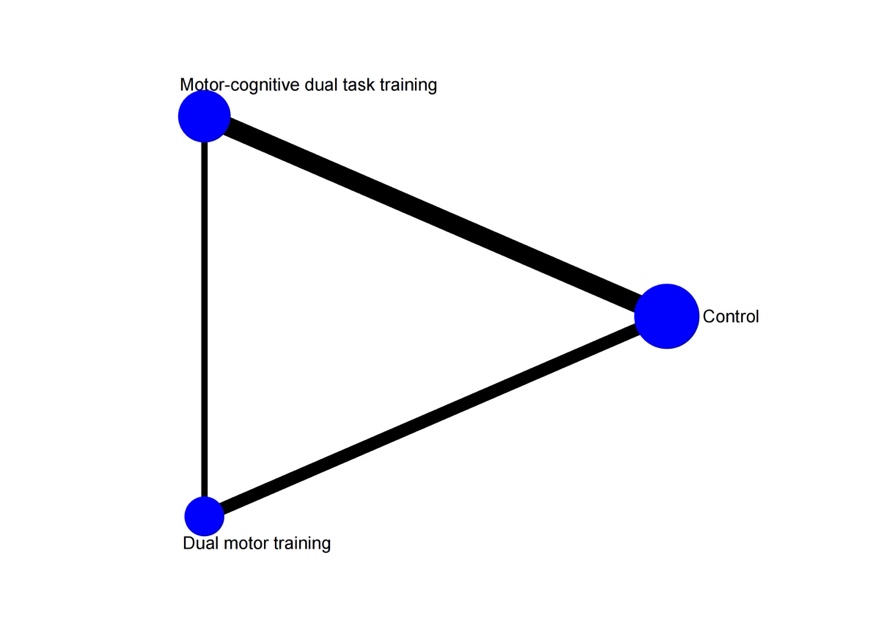


1. Muscle strength


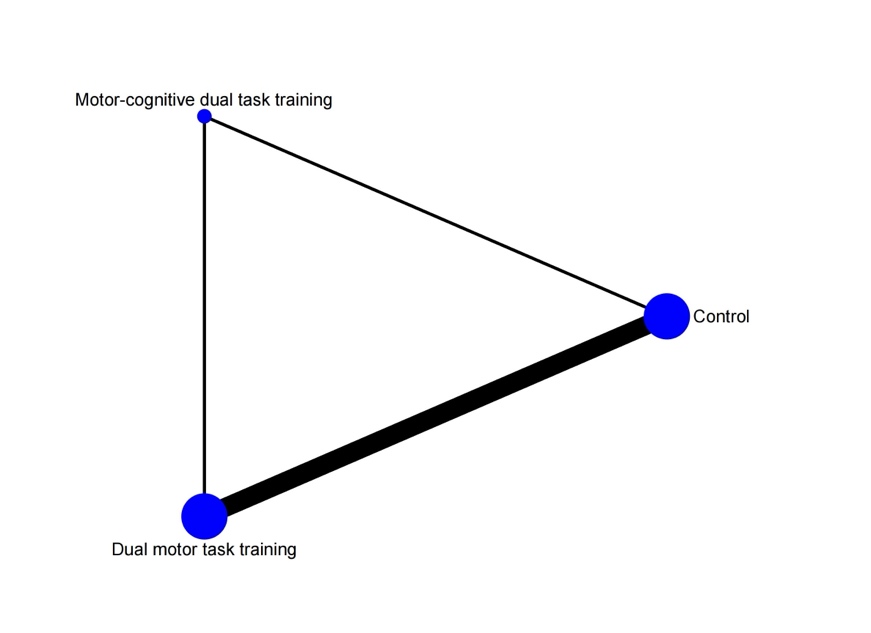


1. Quality of life


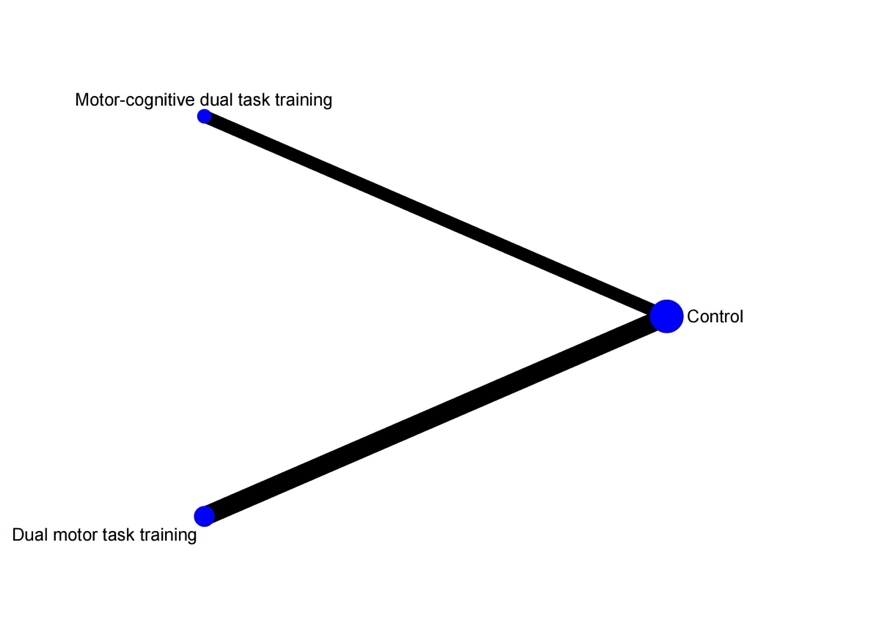


1. Balance


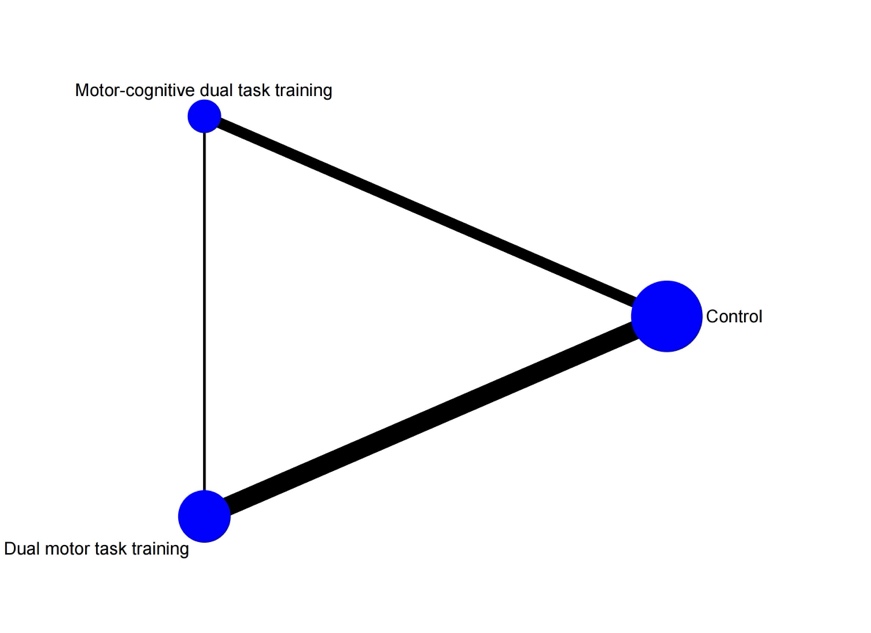


**Supplementary Appendix 7 -- Results of the loop-specific inconsistency tests**

1. Global cognition

1. Executive function

1. Memory function

**Supplementary Table 8 -- Funnel plot for all outcomes**

1. Global cognition

1. Executive function
2. Memory function

1. Depressive symptoms

1. ADL
2. Gait performance
3. Muscle strength

1. Quality of life

1. Balance

**Supplementary Appendix 9 -- Pairwise network meta-analysis results**

1. Global cognition


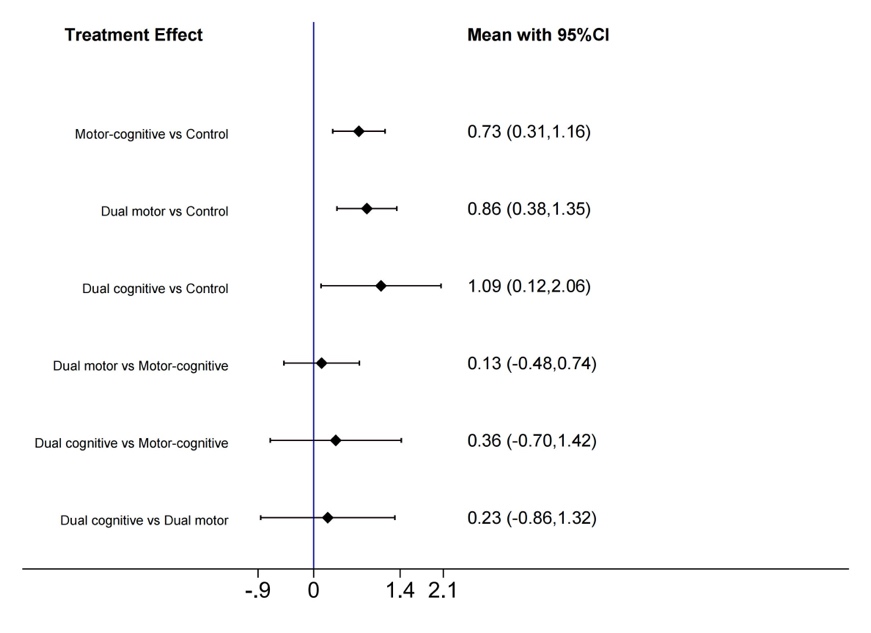


1. Executive function


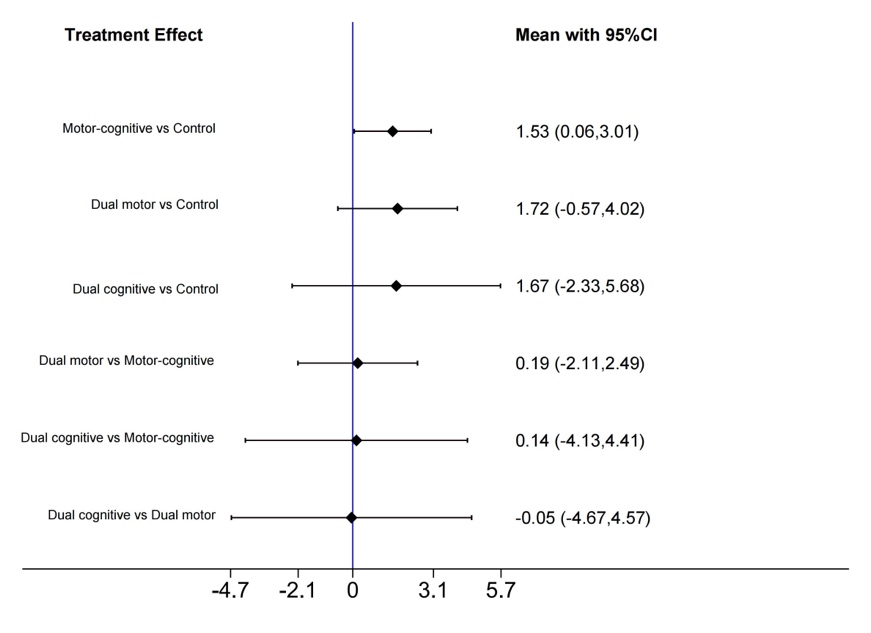


1. Memory function


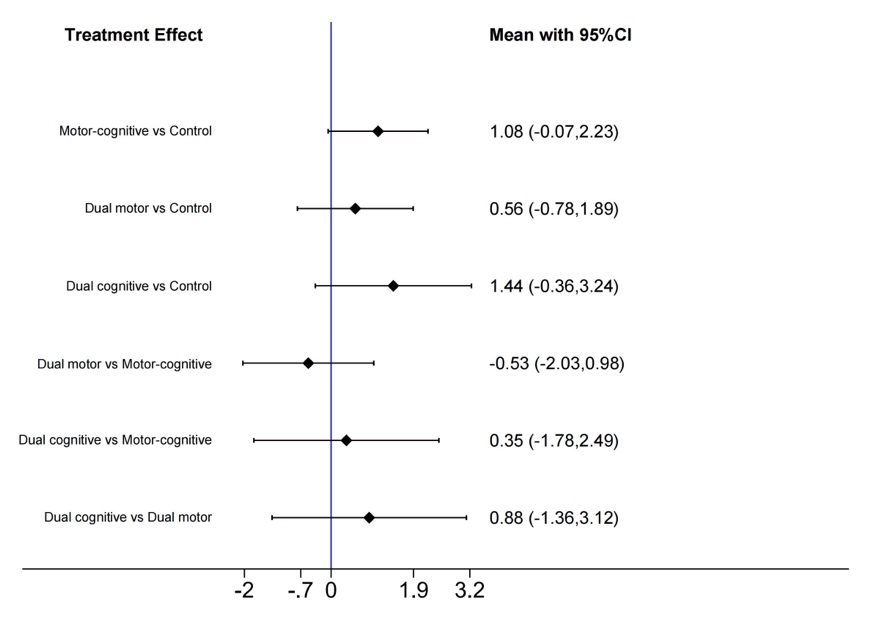


1. Depressive symptoms


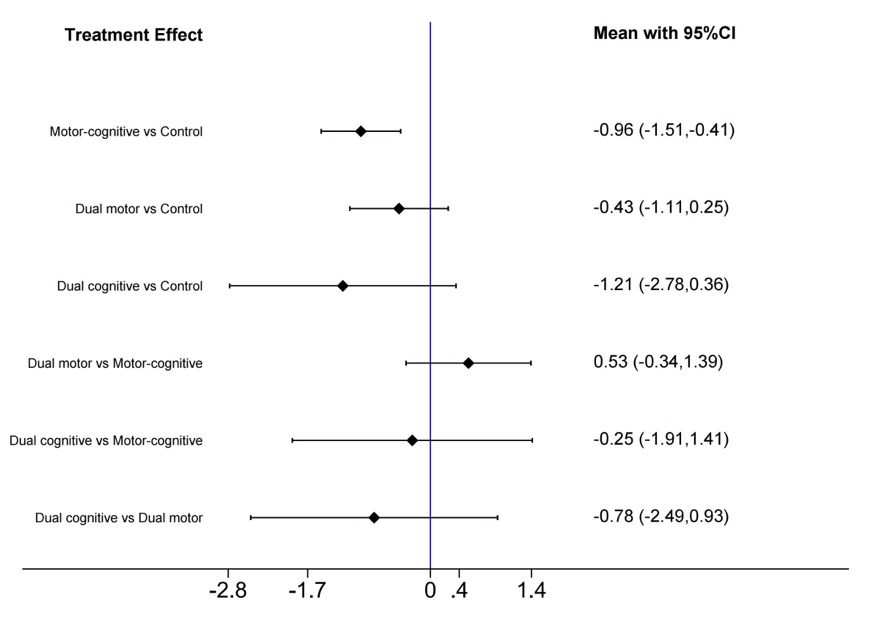


1. ADL

1. Gait performance


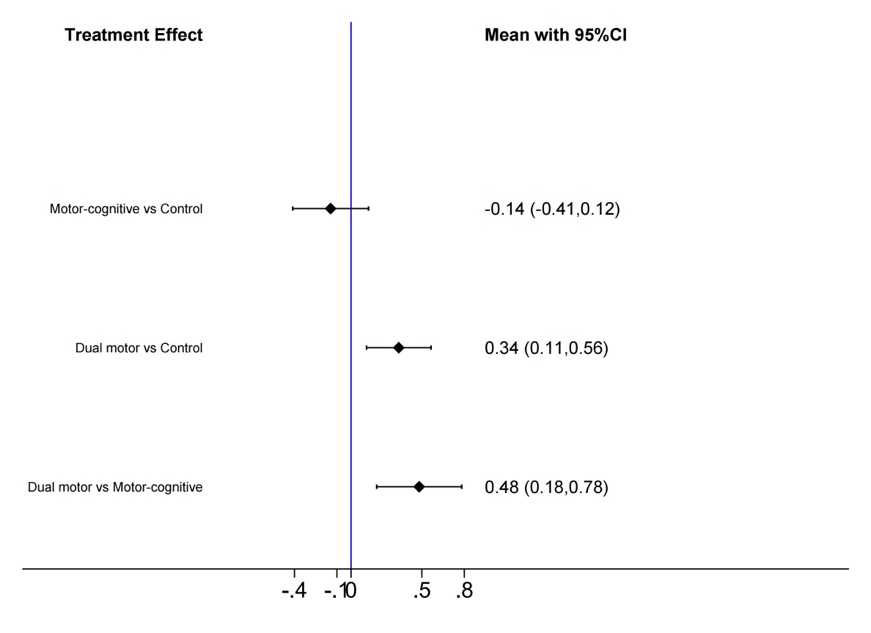


1. Muscle strength


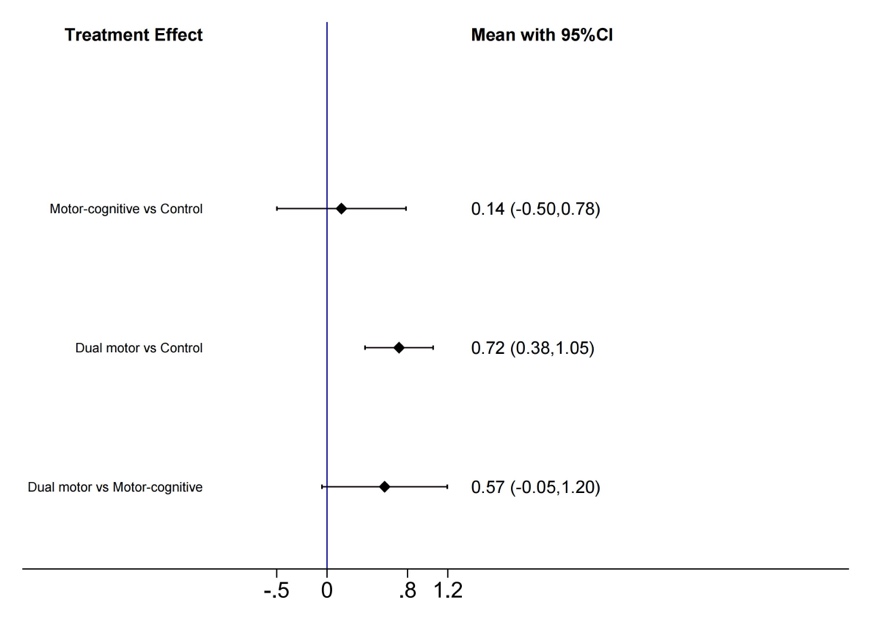


1. Quality of life


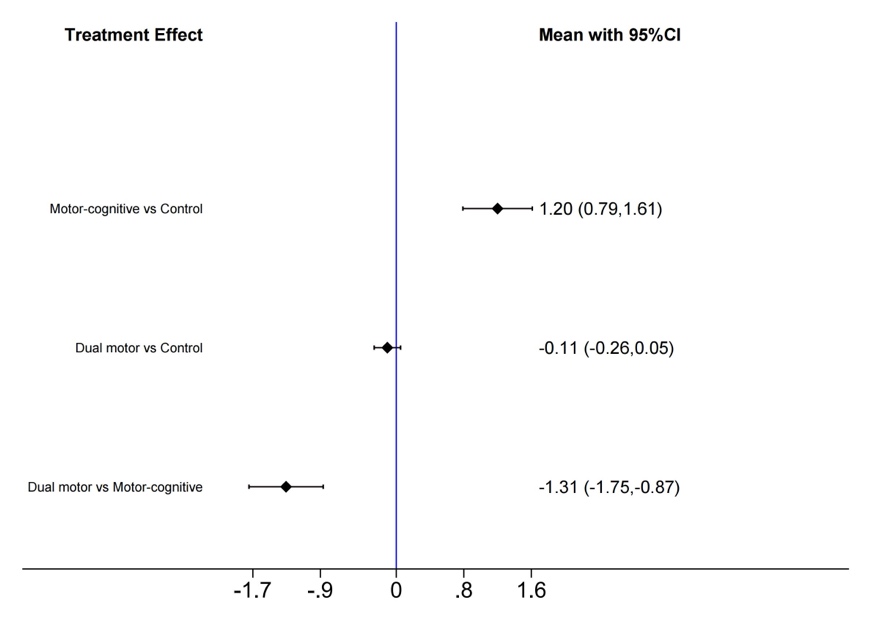


1. Balance


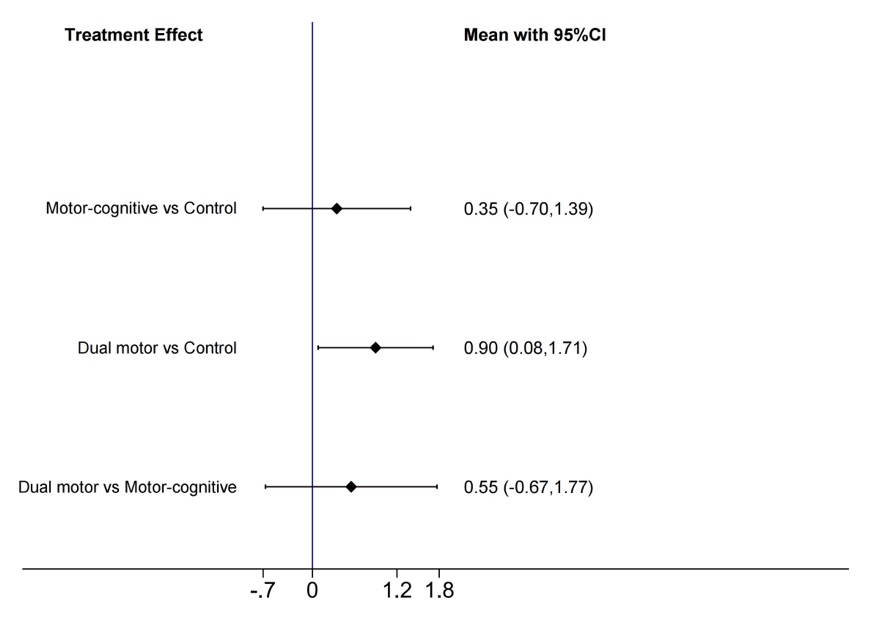


**Supplementary Table 10 -- The ranking of effects for the remaining outcomes：the SUCRAs and the League tables**

1. Depressive symptoms


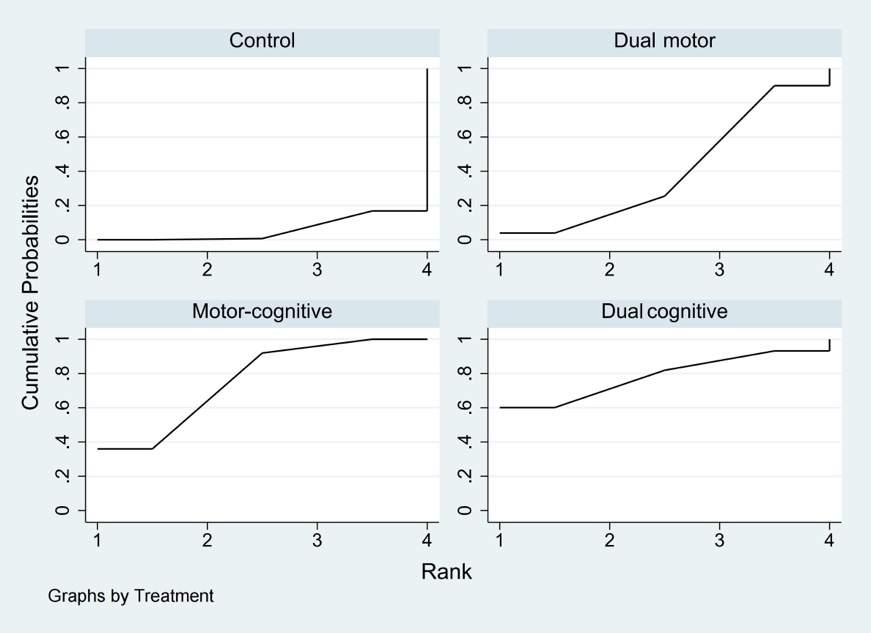


| **Dual cognitive** |  |  |  |
| --- | --- | --- | --- |
| -0.25 (-1.91, 1.41) | **Motor-cognitive** |  |  |
| -0.78 (-2.49, 0.93) | -0.53 (-1.39, 0.34) | **Dual motor** |  |
| -1.21 (-2.78, 0.36) | **-0.96 (-1.51, -0.41)** | -0.43 (-1.11, 0.25) | **Control** |

Note: For Depressive symptoms, a negative SMD indicates a preference for the lower-right intervention; a positive SMD favors the upper-left intervention. This contrasts with other outcomes where a positive SMD reflects a more favorable effect.

1. ADL


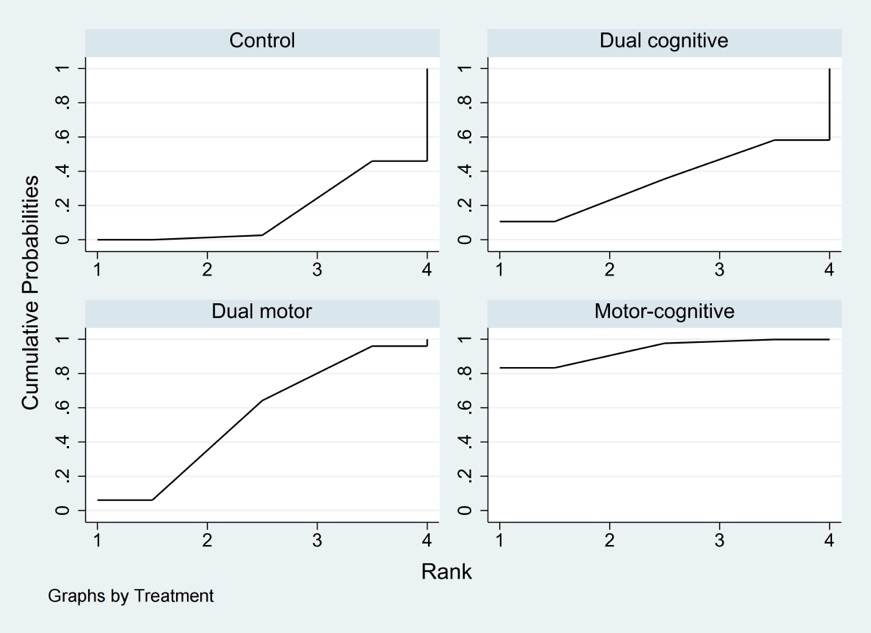


| **Motor-cognitive** |  |  |  |
| --- | --- | --- | --- |
| 0.91 (-0.33, 2.14) | **Motor-cognitive** |  |  |
| 1.29 (-0.88, 3.45) | 0.91 (-0.33, 2.14) | **Dual cognitive** |  |
| **1.50 (0.50, 2.50)** | 1.29 (-0.88, 3.45) | 0.22 (-1.71, 2.14) | **Control** |

1. Gait performance


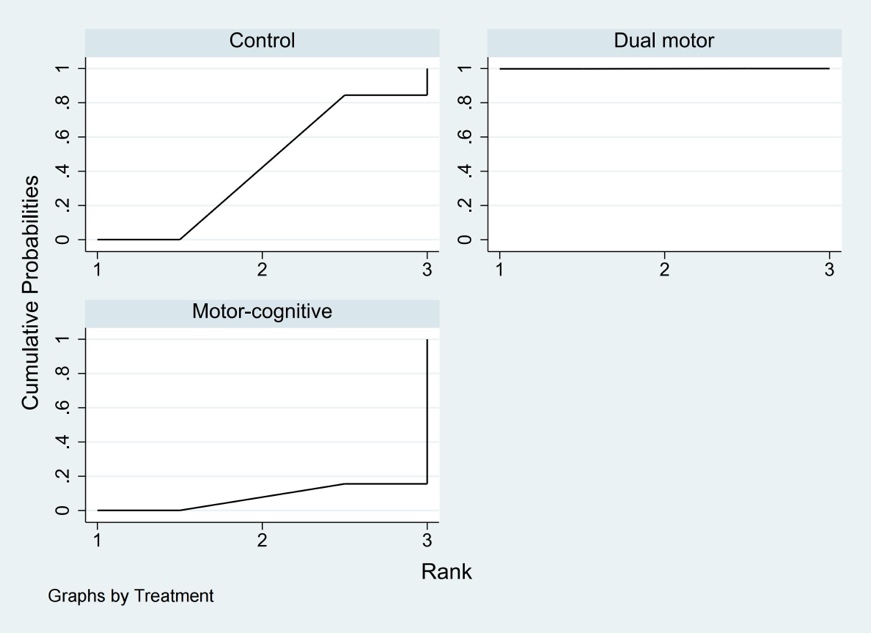


| **Dual motor** |  |  |
| --- | --- | --- |
| **0.34 (0.11,0.56)** | **Control** |  |
| **0.48 (0.18,0.78)** | 0.14 (-0.12,0.41) | **Motor-cognitive** |

1. Muscle strength


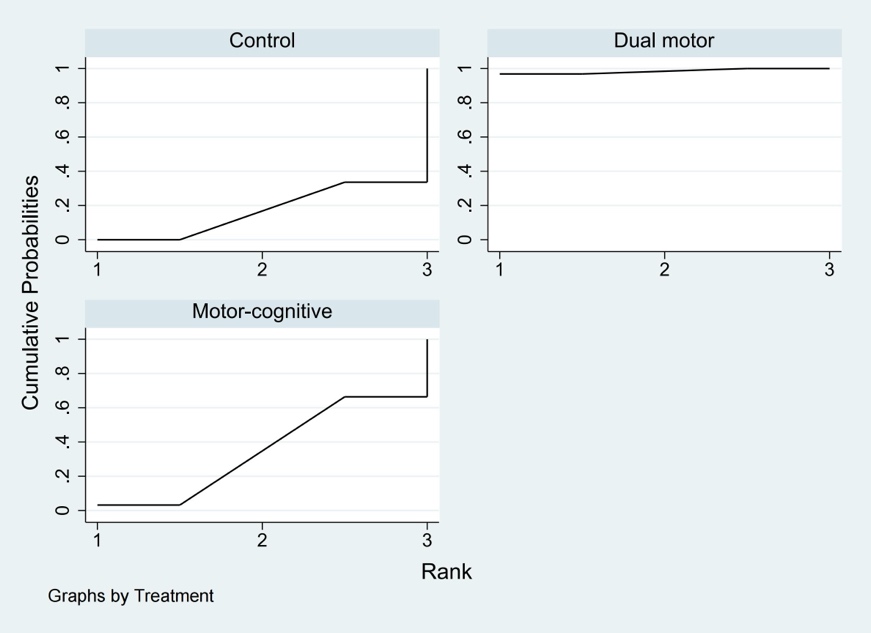


| **Dual motor** |  |  |
| --- | --- | --- |
| 0.57 (-0.05,1.20) | **Motor-cognitive** |  |
| **0.72 (0.38,1.05)** | 0.14 (-0.50,0.78) | **Control** |

1. Quality of life


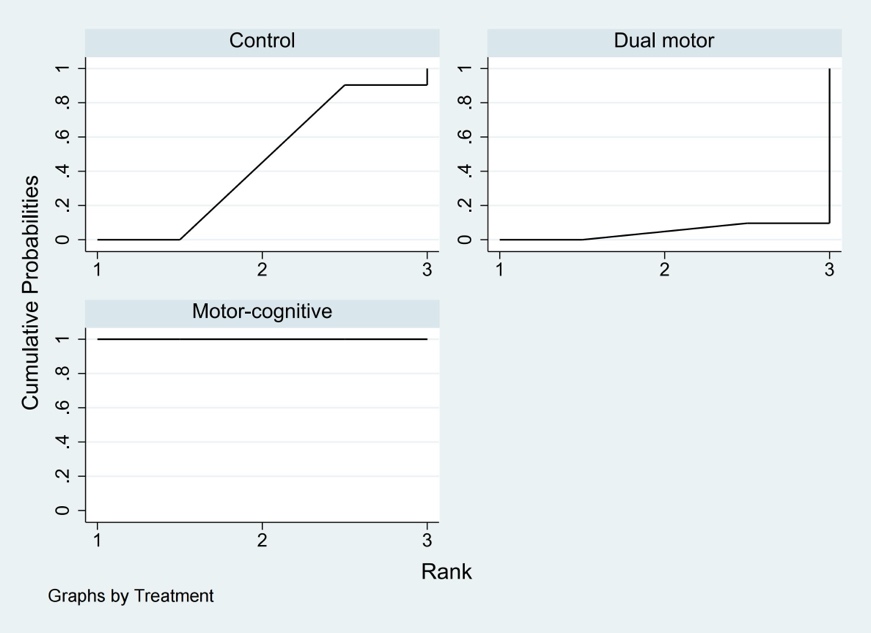


| **Motor-cognitive** |  |  |
| --- | --- | --- |
| **1.20 (0.79,1.61)** | **Control** |  |
| **1.31 (0.87,1.75)** | 0.11 (-0.05,0.26) | **Dual motor** |

1. Balance


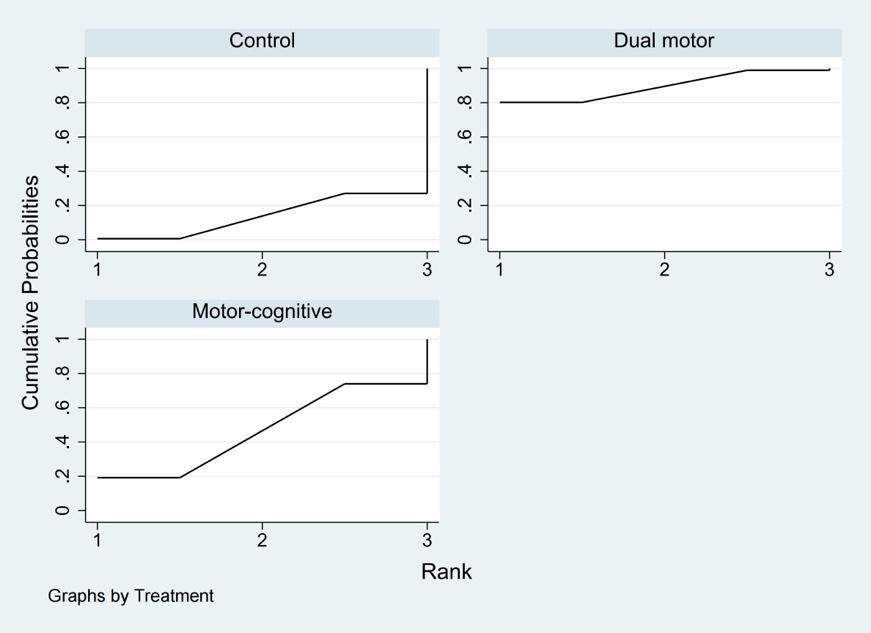


| **Dual motor** |  |  |
| --- | --- | --- |
| 0.55 (-0.67,1.77) | **Motor-cognitive** |  |
| **0.90 (0.08,1.71)** | 0.35 (-0.70,1.39) | **Control** |
